# Amphiphilic Semisynthetic Triterpenoids Impair Survival Pathways and Suppress Clonogenic Growth in Multidrug-Resistant High-Risk Neuroblastoma

**DOI:** 10.3390/ijms27156563

**Published:** 2026-07-23

**Authors:** Silvana Alfei, Cinzia Domenicotti, Sara Tirendi, Elaheh Khaledizadeh, Dafni Graikioti, Constantinos M. Athanassopoulos, Guendalina Zuccari, Caterina Reggio, Barbara Marengo

**Affiliations:** 1 Department of Pharmacy, University of Genoa, Viale Cembrano, 16148 Genoa, Italy; guendalina.zuccari@unige.it; 2 Department of Experimental Medicine (DIMES), University of Genova, Via Alberti L.B., 16132 Genoa, Italy; sara.tirendi@edu.unige.it (S.T.); elaheh.khaledizadeh@edu.unige.it (E.K.); barbara.marengo@unige.it (B.M.); 3 AOM-IRCCS Ospedale Policlinico San Martino, 16132 Genoa, Italy; 4 Inter-University Center for the Promotion of the 3Rs Principles in Teaching & Research (Centro 3R), 56122 Pisa, Italy; 5 Department of Chemistry, University of Patras, University Campus Rio Achaias, 26504 Rio, Greece; dafnigraikioti@upnet.gr (D.G.); kath@upatras.gr (C.M.A.); 6 Laboratory of Experimental Therapies in Oncology, IRCCS Istituto Giannina Gaslini, Via G. Gaslini 5, 16147 Genoa, Italy; caterinareggio@gaslini.org

**Keywords:** high-risk NB (HR-NB), natural triterpenoids, synthetic triterpenes, nanovesicles, cell viability, clonogenicity, Akt and ERK1/2 kinase system

## Abstract

High-risk neuroblastoma (HR-NB) remains a major clinical challenge due to the emergence of therapy resistance. In this study, the anticancer effects of seven previously synthesized betulin (BET), betulinic acid (BA) and ursolic acid (UA) derivatives (**1**–**7**) and of their natural precursors BET, BA and UA (**8**–**10**) were investigated in HTLA NB cells, selected as the experimental model by MTT assay, to find a possible solution to drugs that have lost their effect. Dynamic light scattering (DLS) analysis showed that amphiphilic compounds **1** and **4**–**7** form nanovesicles (240–448 nm) in water, while all compounds have high positive ζ-potential (ζ-p, +28.5–+83.1 mV), supporting favourable membrane interaction and cellular uptake. Cytotoxic experiment results and related IC_50_ values were expressed as the mean ± SD of four independent experiments run in triplicate. Most derivatives exhibited a cytotoxic activity higher than that of their natural precursors and outperformed etoposide; they were particularly effective against the multidrug resistant (MDR) HTLA ER cells. Among them, the ursolic acid (UA) derivative **7** emerged as the most active compound, displaying sub-micromolar to low micromolar IC_50_ values and markedly improving the activity of native UA. Functional studies revealed that it induces complete suppression of clonogenic growth at low micromolar concentrations in both HTLA ER and parental HTLA 230 NB cells. In addition, a concentration-dependent downregulation of Akt, p-Akt, BMI1 and PARP, was observed consistently with a marked suppression of survival pathways and loss of cellular homeostasis. Collectively, our experiments, which need further direct investigation to confirm subsequent assumption, could suggest that compound **7** could kill cancer cells via a non-apoptotic, bioenergetic collapse mechanism. All of these findings suggest compound **7** as a promising mitochondria-targeted lead candidate and support amphiphilic triterpenoid derivatives as attractive platforms for overcoming multidrug resistance in high-risk NB.

## 1. Introduction

High-risk NB (HR-NB) is one of the most aggressive paediatric extracranial solid tumours and it is characterised by amplification of the MYCN proto-oncogene [1,2]. Despite advances in multimodal therapeutic strategies, including intensive chemotherapy, radiotherapy, surgery, and immunotherapy, the prognosis of HR-NB patients remains poor, with survival rates below 50% [3,4,5,6], due to the development of chemoresistance, which promotes tumour relapse and therapeutic failure. Combined therapies including etoposide (ETO), doxorubicin, cisplatin, vincristine, etc., initially give promising outcomes, later annihilated by NB relapses and acquired drug resistance, as demonstrated and reported in our previous works [3,6].

In this regard, the use of a combinatorial treatment approach could only delay the onset of resistance and improve patient outcomes. In any case, undesired drug–drug interactions could negatively affect the therapeutic effects [7]. Therefore, the search for compounds with an extra-genomic mechanism of antitumor action is of paramount importance to avoid the possible recurrence of cancer and or the emergence of resistance.

Within this context, mitochondria are the most promising target for an effective extra genomic anticancer therapy due to their key role in energy production, apoptosis induction, and ROS generation [8]. Recent studies have shown that several metastatic, therapy-resistant, and cancer stem cells are reliant on mitochondrial respiration and upregulate oxidative phosphorylation (OXPHOS) activity to maintain tumorigenesis [9]. Mitochondria are crucial for tumour proliferation, survival, metastasis, and the development of resistance to chemotherapy and radiotherapy. Recent studies report that mitochondria are a vulnerable target for cancer therapy [9]. Additionally, these intracellular organelles possess their own DNA, independently and not associated with the mutable genetic mechanisms of cells [10]. Therefore, mitochondria-targeting compounds, that are capable of accumulating inside this organelle and impairing its functions, would succeed in bypassing the genetic mechanisms underlying tumour recurrence and resistance, thus also being active in resistive cells.

The consequent inhibitory activity on the mitochondria’s functions determines mitochondrial toxicity and permanent damage, thus causing the programmed death of cancer cells by the signal induction of cell death processes (apoptosis, necroptosis, or autophagy) [11]. In the present study, a panel of semisynthetic triterpenoids and their natural precursors were evaluated in HTLA ER [6] cells and drug-sensitive HTLA 230 cells, to identify novel compounds with potential activities against both phenotype, but especially MDR HTLA ER. The most effective molecule was subsequently selected for more in-depth investigations on its capability to suppress clonogenic growth and possible mechanisms of actions of our compounds. Anyway, the study hypothesis and primary aims are detailed in the subsequent Section “The Rationale of This Study”.

### The Rationale of This Study

**Scheme 1:**
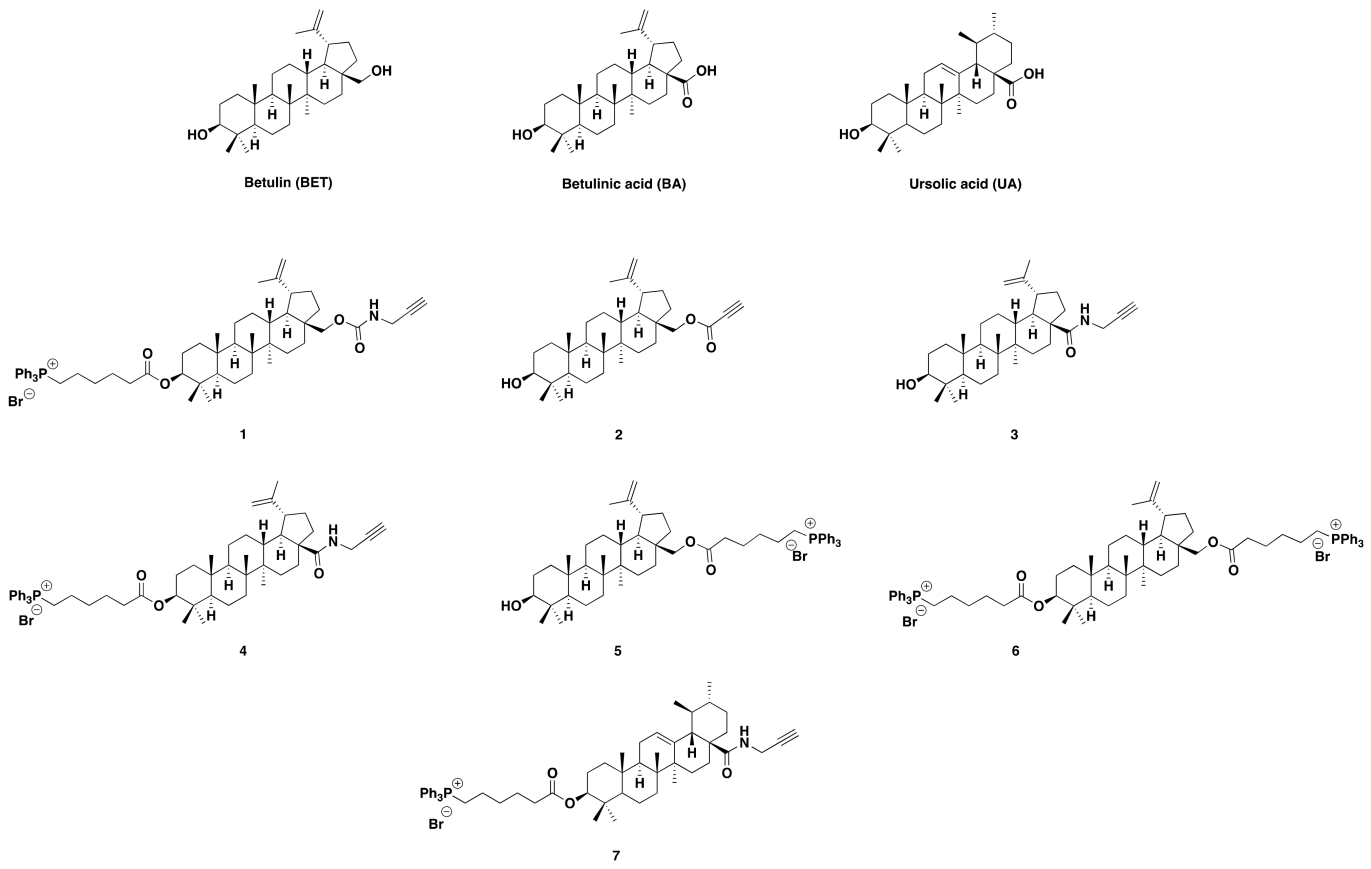
Chemical structure of synthetic triterpenoids **1**–**7** recently synthetized and natural compound BET, BA and UA (compounds **8**–**10** in this study) used as their naturally occurring *core*. Scheme 1 has been edited using ChemDraw Ultra 7.0.1, a commonly used software to draw chemical structures, globally recognized.

Considering the urgent need for novel therapeutic strategies capable of overcoming MDR in HR-NB, this study aimed at the identification of compounds with enhanced cytotoxic activity (possibly IC_50_ ≤ 10 µM) against drug-sensitive (HTLA 230) and MDR (HTLA ER) cells. Additionally, we aimed to identify molecules displaying higher efficacy than that of etoposide (ETO), whose activity is significantly reduced in chemo-resistant NB cells [6,12]. Our hypothesis was that the identification of such compounds could offer new therapeutic opportunities, especially for patients refractory to currently available drugs such as ETO, paclitaxel and DOX [11,13,14]. The basic idea for this study was that, among natural compounds with emerging anticancer potential, triterpenoids attract considerable attention. In fact, they are capable of modulating multiple cellular processes involved in tumour progression, including the oxidative stress (OS) response, apoptosis, and metabolic adaptation. Particularly, betulin (BET), betulinic acid (BA) and ursolic acid (UA), have shown promising antiproliferative and pro-apoptotic effects in several cancer models. By a literature review, several approaches have been found to create ionic platforms based on triterpenoids. Systems possessing salt-like or amphiphilic structures, such as quaternary ammonium, pyridinium, or triazolium salts of triterpenoids, have been developed. Additionally, a conceptually new direction, regarding the construction of binary ionic compounds, in which triterpene fragments simultaneously act as both a cation and an anion, have also been reported [15,16,17,18,19,20,21,22]. On a related note, we have recently synthesised and characterized seven semi-synthetic triterpenoid (**1**–**7**) [23] derivatives generated through selective chemical modifications at the C-3 and/or C-28 positions of BET, BA and UA scaffolds [23] (Scheme 1).

These compounds demonstrated considerable antibacterial properties, mainly due to the presence of cationic alkyl triphenyl amphiphilic phosphonium (TPP^+^) groups, linked through a lipophilic alkyl chain [23]. TPP^+^ groups are known to induce membrane damage and cellular uptake, due to their amphiphilic and positively charged nature [11,13]. Interestingly, several studies [11,13,14,15,16,17,18,19] reported that cationic compounds of several types, including quaternary phosphonium salts containing TPP^+^, can also exert selective anticancer activity [14,24]. Typically, this is due to easy electrostatic interactions with the negatively charged constituents of the cancer cell plasma membrane. This event leads to membrane depolarization and irreversible damage, with consequent cellular uptake, mitochondrial accumulations, energy homeostasis disturbance, and ultimately cell death [25]. Since these mechanisms are largely independent of direct genomic targeting, cationic molecules may represent promising candidates for overcoming multidrug resistance. Based on these observations, antibacterial compounds **4**–**7**, bearing the cationic TPP^+^ group inserted by a lipophilic C-6 chain, appeared to be excellent candidates for our objective. In parallel, compounds **2** and **3**, despite not bearing the alkyl TPP^+^ group, were included in our arsenal, because **2** reproduced the structure of 28-*O*-propynoylbetulin (EB5) and **3** was very similar to 28-*O*-propargyloxycarbonylbetulin (EB25/1), with a BA *core* in place of BET. EB5 and EB25/1 showed anticancer activity in NB cells through inhibition of the Akt and Erk kinases system, as reported by Król et al. [26]. These signalling pathways, involved in cell survival, anti-apoptosis mechanisms, proliferation, and damage repair, are frequently even upregulated in chemo-resistant tumours [27,28,29,30,31,32]. Furthermore, compound **1** was also considered of particular interest because it contains the BET *core* as EB5, the C-6 alkyl TPP^+^ group on C-3, and the Akt-inhibitor propargylamine moiety as EB25/1. Also, the combination of natural components, such as BET, never reported to exert toxic effects on healthy human cells, as well as BA and UA, reported to be cytotoxic on healthy cells only at relevant concentrations or under conditions that alter autophagy [33,34], with known anticancer groups, appeared as a good choice to obtain low cytotoxicity levels against healthy cells. We recognise as a limitation of this study the absence of experiments in non-tumoral cells, and absence of direct mitochondrial functional assays. Anyway, these additional experiments were not in the scope of this already articulated paper, whose specific aims have been previously reported. As also clarified in several other parts of the paper, other compounds in addition to compound **7** are also worthy of more in-depth biological assays, for the moment not carried out, but that surely will be performed soon. Embedding a single work with too much data can lead to confusion and misunderstanding. The same can be applied to missing experiments, which will be undertaken soon.

## 2. Results and Discussion

### 2.1. Semi-Synthetic Triterpenoids ***1***–***7*** Possessing a Betulin (BET), Betulinic Acid (BA) and Ursolic Acid (UA) Core

The seven semi-synthetic triterpenoids (**1**–**7**) were previously prepared by chemically decorating BET (**8**), BA (**9**) and UA (**10**), used as natural triterpenoid *cores*, via different linkages of the acetylene, propargyl and/or 6-TPP^+^-hexanoic acid moieties, on the hydroxyls in C-3 and/or C-28 carbon atoms of *cores* [23]. Their detailed synthesis and chemical characterization are available in a recent paper [23].

### 2.2. The Roadmap of Experiments in the Project

**Scheme 2:**
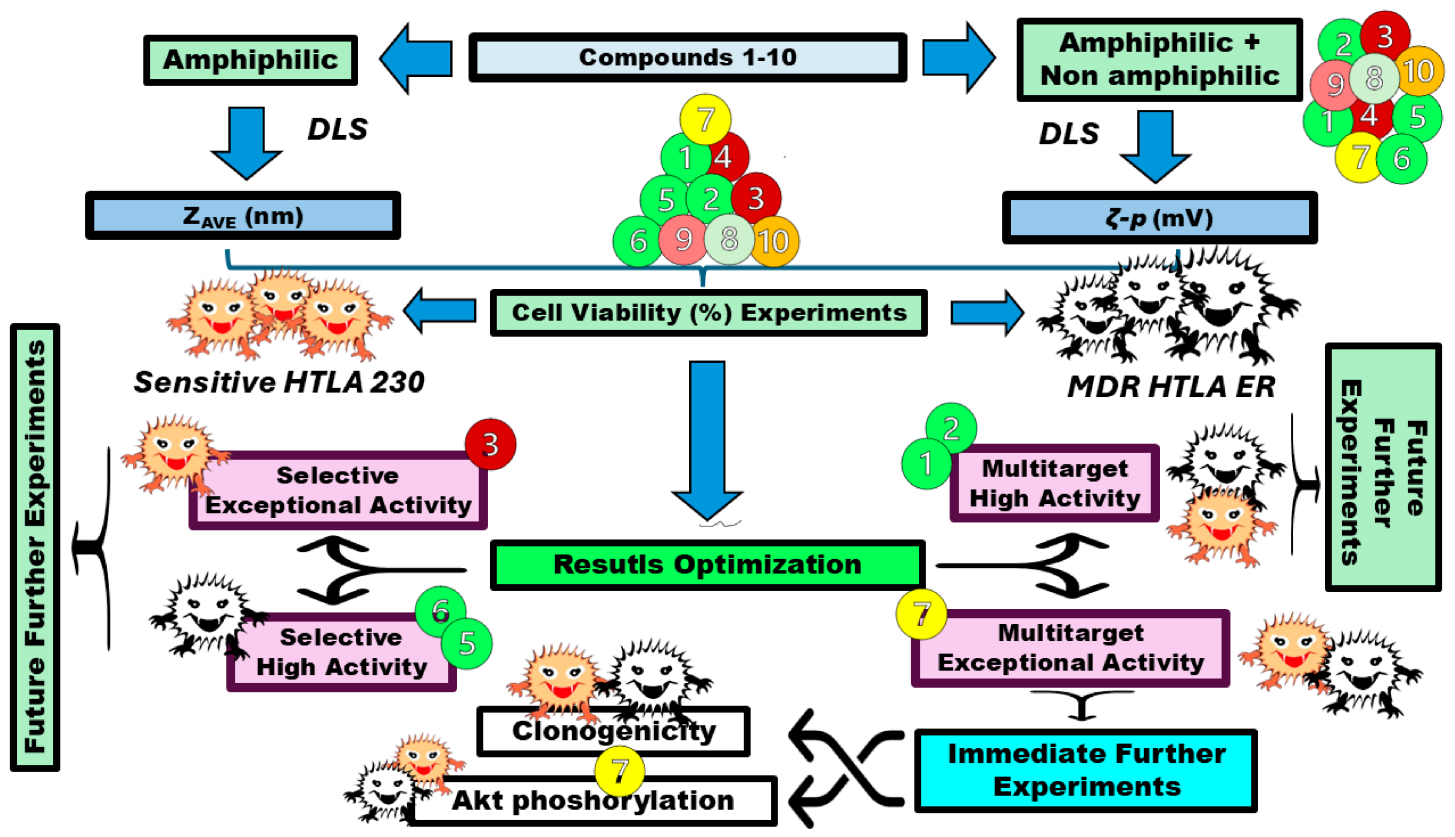
Roadmap followed to investigate synthetic and natural triterpenes **1**–**10** and indications for compounds which will be investigated further. The Scheme is home-made by Authors using Microsoft 365, PowerPoint software and related tools.

Scheme 2 shows the roadmap followed for the experiments in this study and indications for the compounds which will be investigated further.

Following the rationale described in Section “The Rationale of This Study”, compounds **1** and **4**–**7**, having amphiphilic characteristics, were first investigated by dynamic light scattering (DLS) technique to assess their behaviour in water solution, in terms of forming nano-vesicles, and to measure their hydrodynamic diameter (nm). In the meantime, the ζ-p values (mV) of all compounds (**1**–**7**) were investigated, since both surface charge and supramolecular organization could have great influence on the interaction of bioactive molecules with cancer cell membranes as well as their cellular uptake [35,36]. Subsequently, the cytotoxic activity of semi-synthetic triterpenoids **1**–**7** and of the corresponding natural triterpenoids **8**–**10**, used as reference compounds, was tested in drug-sensitive and MDR NB cells. Cell viability was assessed by MTT assay after 24, 48 and 72 h of treatment, and the most representative results are reported in Scheme 1. Among the tested molecules, except for compound **4**, **1**–**3** and **5**–**7** compounds were all found to be particularly interesting, each of them for specific applications against NB cells and worth further in-depth investigation. Anyway, since compound **7** displayed the strongest multitarget and most consistent cytotoxic activity, thus immediately meeting all of our aims, it was here selected for completing this study with further mechanistic insights, through clonogenic assays, aimed at evaluating the ability to form colonies. Finally, since the Akt pathway is associated with NB progression and drug resistance, its phosphorylation status was analysed in both NB cell populations before and after treatment with compound **7**, to evaluate the possible involvement of this pathway in the biological effects exerted by the compound.

### 2.3. Dynamic Light Scattering (DLS) Analyses

*Bis*-triphenyl phosphonium (TPP^+^) compounds having two TPP^+^ cationic heads, linked by hydrophobic carbon chains and defined as bola-amphiphiles, spontaneously self-assemble into spherical vesicles [37,38], when dispersed in aqueous solution at the proper concentration. Such vesicles appeared micro-dimensional under the optical microscope and nano-dimensional via the DLS analysis, with positive ζ-potential (ζ-p) [39,40,41]. Based on these data, compounds **1** and **4**–**7** of this study, all having the C6-alkyl-TPP^+^ group, capable of conferring amphiphilic characteristics, due to the cationic phosphonium head and the C6 alkyl chain, were analysed by DLS technique to assess their capability to form nanovesicles, as well as to measure their hydrodynamic diameter and PDI. Moreover, with the same instrument, all semisynthetic compounds **1**–**7** were analysed for assessing their zeta-potential (ζ-p, mV) and surface charge. ζ-p is linked to the stability of particle solutions/dispersions [42,43] and to their possible capability to interact with the negatively charged surface of cancer cells and to kill tumour cells by membrane damage and secondary events [44,45]. Generally, higher values of ±ζ-p forecast higher stability [43], while higher values of +ζ-p correspond to a higher possibility of interaction with negatively charged constituents of cancer cell membranes (lipopolysaccharides, teichoic acids, and anionic phospholipids), by positive surface charge, proportionally to ζ-p values [44,46,47]. Stronger interactions, such as those promoted by quaternary alkyl TPP^+^ salts (QPSs), translate into stronger adhesion, with consequent irreversible impairments, surface charge neutralization, destabilization of the lipid bilayer, pore formation, and increased membrane permeability [47]. Such events result in either rapid loss of cell integrity and collapse, or in providing access to positive TPP^+^ compounds inside the cell, where they can accumulate in negative districts, such as mitochondria, whose membrane potential is even more negative (−220 mV) than that of mitochondria of normal cells (−160 mV) [48,49], with lethal impairment of their vital functions. In mitochondria, TPP^+^ molecules can interfere with mitochondria functions, inhibiting glycolysis, depolarizing the membrane potential, and inhibiting the mitochondrial permeability transition pore, thus leading to cancer cell mitochondria disruption. These events kill cancer cells mainly by apoptotic death, thus making TPP^+^ QPSs, mitochondria-targeting therapeutic agents [49]. The mitochondria-mediated anticancer effects of TPP^+^ QPSs are a further reason why TPP-containing QPSs having high affinity for mitochondria are multitarget weapons that are also active against bacterial superbugs [23,37,39], which, as is known, are their ancient parents, from which mitochondria have evolved [50]. Concerning dimensional distribution of **1** and **4**–**7**, three measurements, made of several runs (records) for each one, were carried out. Demonstrative images of single or multiple records acquired by number (%) are reported in Section S1 of the Supplementary Materials, shown in Figures S1–S6. Dimensions and polydispersity index values (PDIs) of all compounds tested are reported in Table 1.

Specifically, the reported Z_AVE_ (nm) in Table 1 is the quantitative mean ± S.D. of three independent determinations, at the highest kcps value, with each one made of 10–12 runs, as reported in Experimental Section 3. The PDI related to such records was also provided (Table 1). Additionally, ζ-p measurements for all samples (**1**–**7**), obtained by three measurements, made of 12 records for each one, provided average ζ-*p* values, which were reported as mean ζ-*p* ± S.D in the last column of Table 1. Representative single record images of ζ-p (mV) distribution are available in Section S1.1 of the Supplementary Materials, in Figures S1–S3. Collectively, all compounds analysed were capable of forming polydisperse nanovesicles in water with Z_AVE_ in the range of 240–448 nm and PDI in the range of 0.253–1.000, with compounds **4** and **5** being more polydisperse and having larger particles (448 nm, PDI 1.000 and 386 nm, PDI 0.704), while compound **7** was less polydisperse (0.253 PDI) and had smaller vesicles (240 nm), thus meeting the dimensions of the particles (200 nm) which, empty or drug loaded, demonstrated the best anticancer activity compared to larger (300 nm) and smaller (100 nm) particles [51]. Specifically, among three sizes, 200 nm showed the best efficacy, establishing that a size of 200 nm can allow the highest uptake in cells and increased drug delivery to the cancer cells. Anyway, larger particles (300 nm), reported by Tarasi et al., with dimensions close to those of **1** (253 nm) and **6** (276 nm), also showed appreciable uptake in cells and efficacies of 68% [51]. Based on the abovementioned literature reports, and on the positive ζ-p values > 20 mV observed for all compounds, a good to high possibility that they possess selective cytotoxic effects on cells with highly negative surfaces can rationally be hypothesized for all compounds. It has been reported that, markedly positive ζ-*p* values (+18.3–+57.6 mV) were associated with strong anticancer properties, even at lower concentrations [37,52]. Additionally, it has been reported that compounds with ζ-*p* values from +40 to +60 mV, as with **1**, **4** and **6**, demonstrated good stability in solution, while those with ζ-*p* > 60 mV, as with **5** and **7**, displayed excellent stability, pointing to their potential for systemic administrations.

### 2.4. Effects of Compounds ***1***–***10*** on HTLA Cells

The seven semisynthetic triterpenoids (**1**–**7**) [23] shown in Scheme 1, were tested against HTLA 230 and ER cells for 24, 48 and 72 h, using the MTT assay. Natural triterpenoids BET, BA and UA (**8**–**10**, Scheme 1), which were the *cores* of **1**–**7**, were tested in the same experimental conditions to allow comparison between natural and semisynthetic derivatives and with related triterpenes previously investigated in NB. All determinations were expressed as quantitative means ± S.D. of four independent experiments run in triplicate, as specified in the caption of the figure. Preliminary experiments were initially performed using all compounds (**1**–**10**) within the concentration range 1–100 µM. Cell viability profiles are reported as bar graphs with related statistical significance in Figure S7A–T, of Section S2.1, in the Supplementary Materials.

Compounds **1**, **2** and **3** showed changes in cell viability characterised by fluctuations under some experimental conditions (Figure S7A–F). Conversely, compounds **4**–**10** showed a reproducible and coherent dose–response trend in both cell populations. Specifically, compounds **4**–**6**, in HTLA ER cells, already displayed a marked cytotoxicity at low concentrations (≥5 µM) and remained relatively stable at higher doses, whereas HTLA230 cells showed a more concentration-dependent response (Figure S7G–L; Supplementary Materials). Compound **7** demonstrated a trend like that observed for **4**–**6** on HTLA ER and on HTLA 230 (Figure S7M,N), while natural triterpenoids (**8**–**10**), displayed against both cell lines poor, slightly time-dependent and significantly dose-dependent cytotoxicity (Figure S7O–T). In this regard, it is reported that the MTT assay can become unstable, fluctuating, and unreliable once a certain concentration threshold is exceeded, especially when wide concentration ranges are used [53]. In these cases, reducing the range below the critical threshold could improve data quality, for chemical, physical, and instrumental reasons [53]. The issue arises because MTT does not directly measure cell viability, but rather the residual metabolic capacity to reduce tetrazolium into formazan. When the treatment concentration exceeds a threshold, the reduction system collapses or becomes non-linear, generating noise, artifacts, and false negative or false positive results [53].

**Figure 1:**
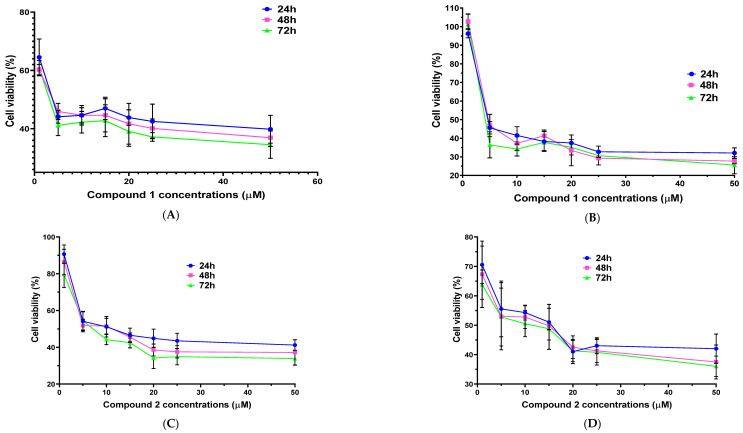

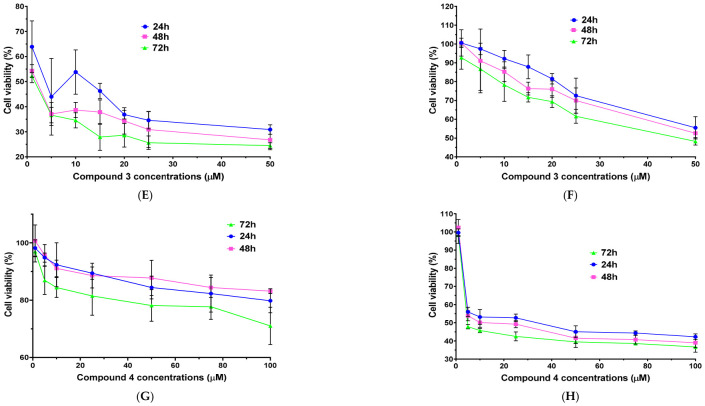

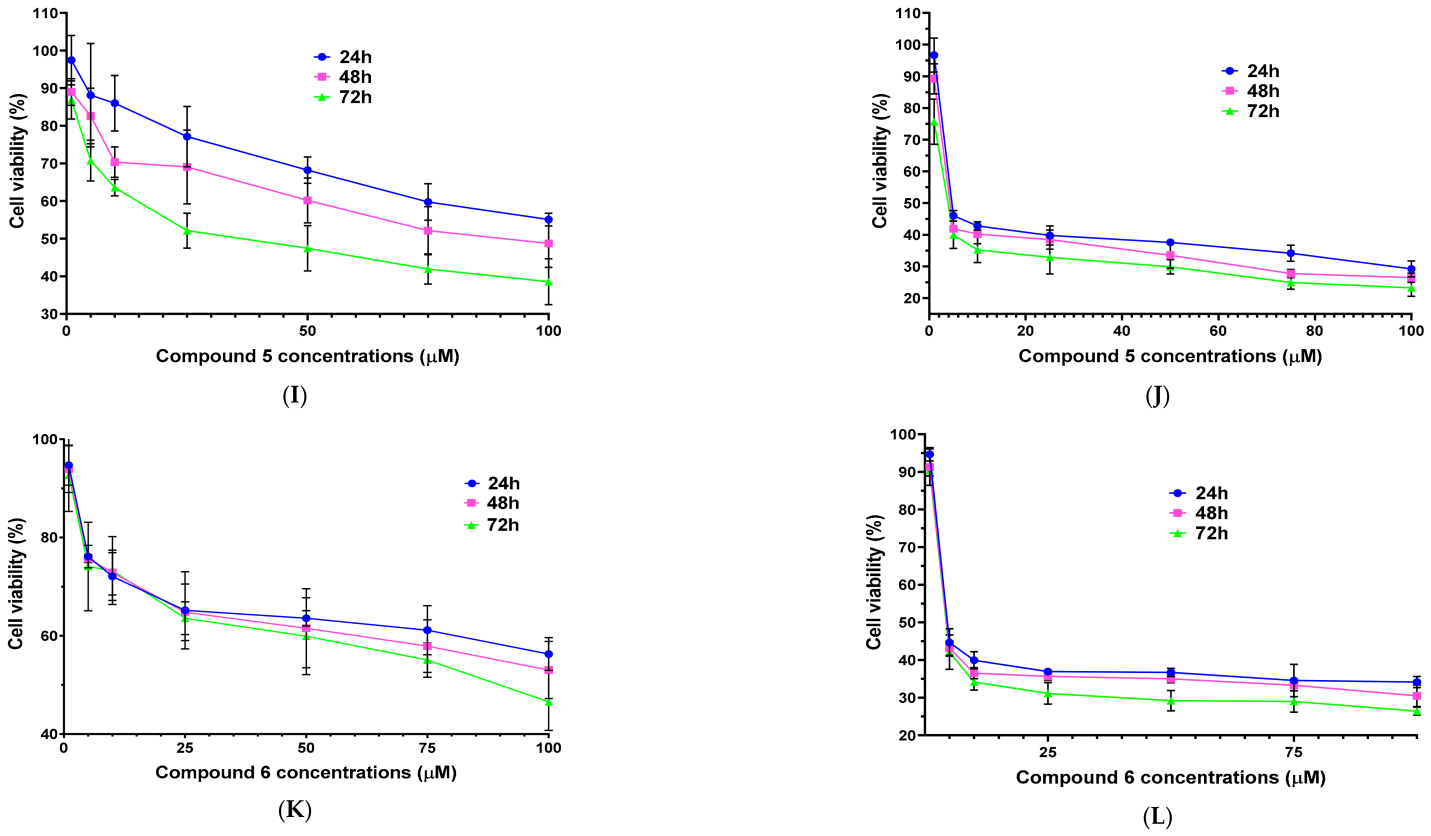

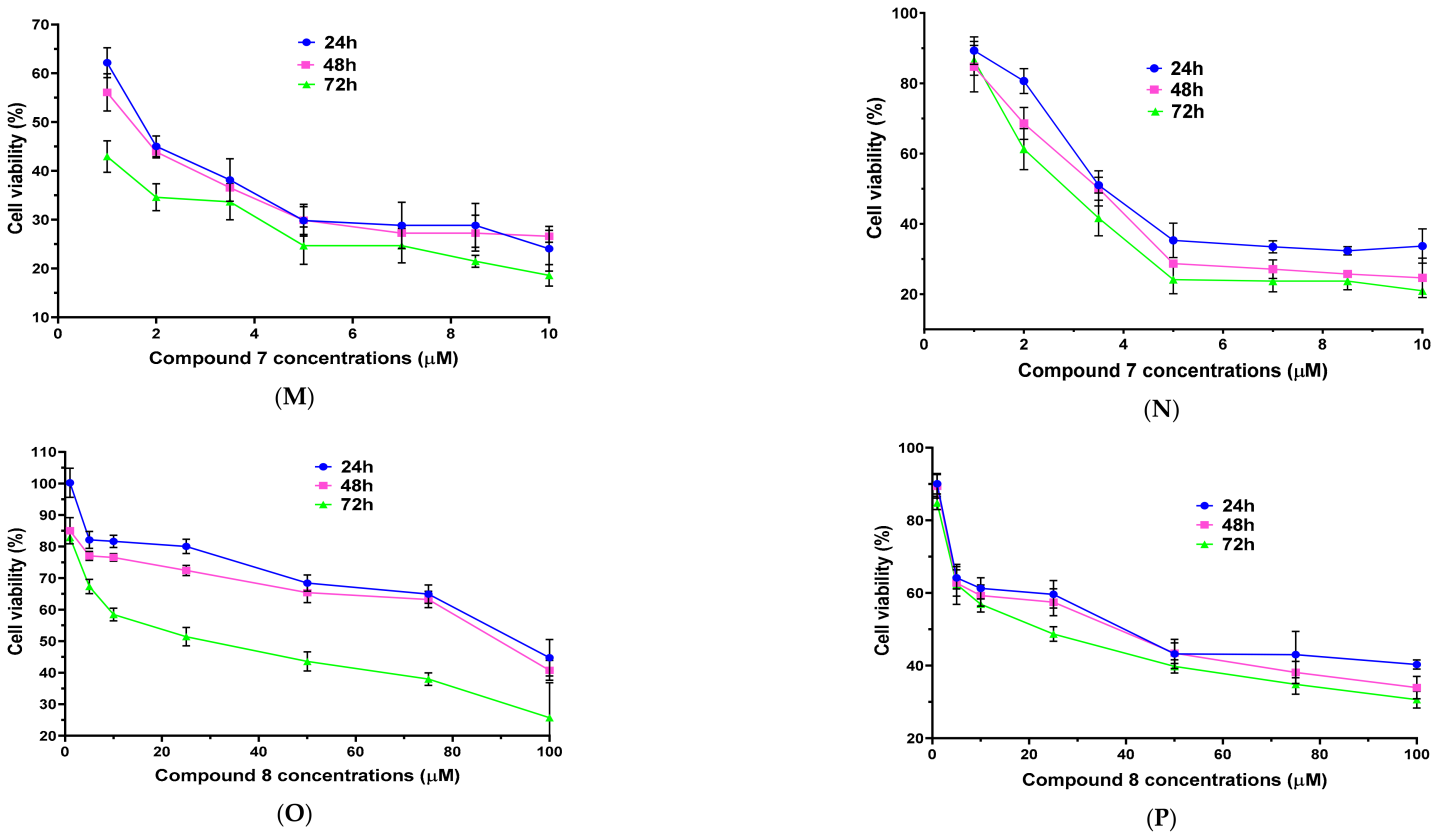

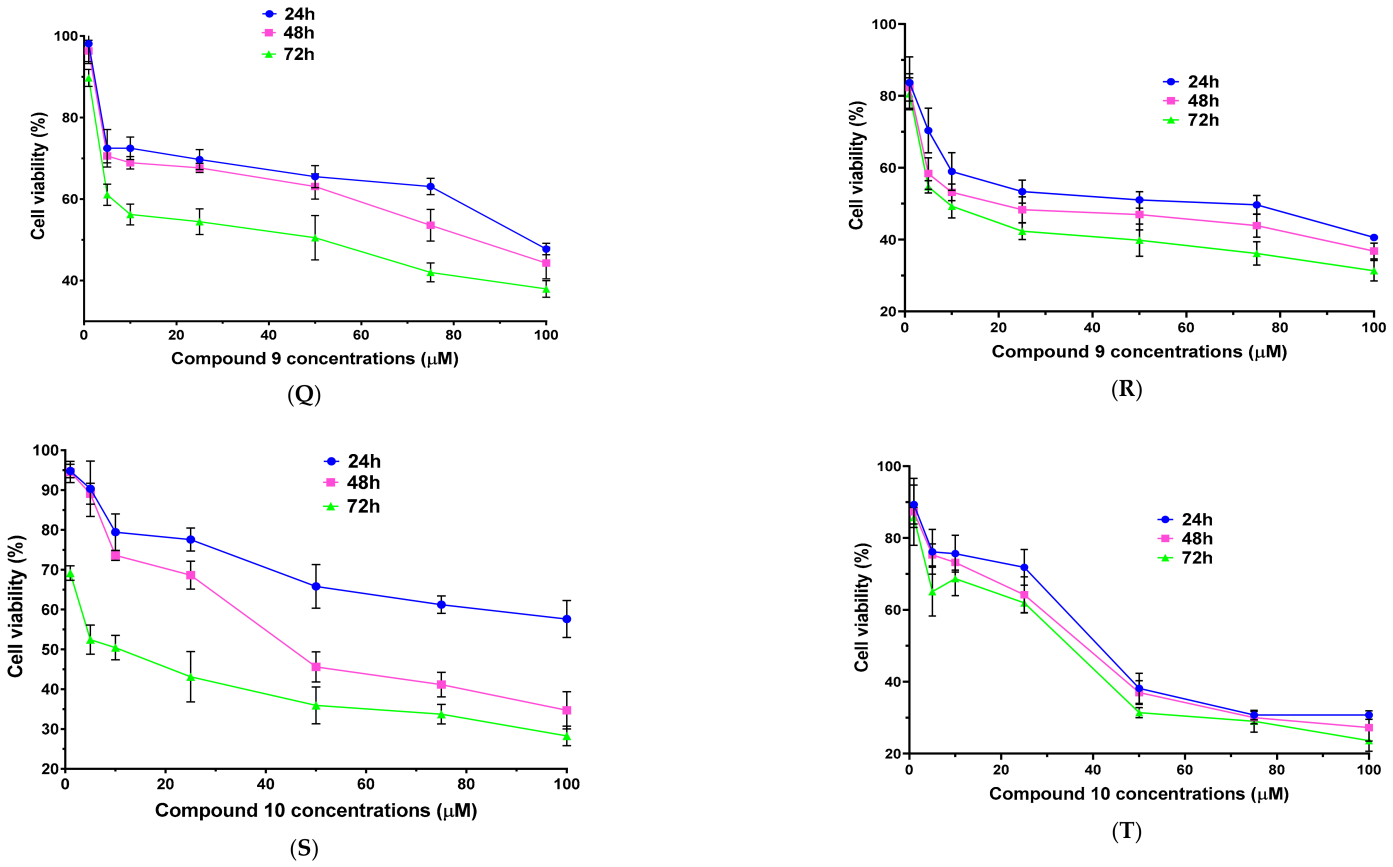
Cell viability was evaluated in HTLA 230 (**A**,**C**,**E**,**G**,**I**,**K**,**M**,**O**,**Q**,**S**) and HTLA ER (**B**,**D**,**F**,**H**,**J**,**L**,**N**,**P**,**R**,**T**) HR-NB cells exposed to increasing concentrations of **1** (**A**,**B**), **2** (**C**,**D**), **3** (**E**,**F**) (1–50 µM), of **4** (**G**,**H**), **5** (**I**,**J**), **6** (**K**,**L**), **8** (**O**,**P**), **9** (**Q**,**R**), **10** (**S**,**T**) (1–100 µM) and **7** (**M**,**N**) (1–10 µM), for 24, 48 and 72 h. Each dispersion graph summarizes quantitative data of the means ± S.D. of four independent experiments run in triplicate. The scale on the x-axis is different for compounds **1**–**3** and **7**, with respect to the others, as a result of optimized experiments. Significance has not been reported in these images, since it has already been reported in bar graph images (Supplementary Materials, Figures S7, S8 and S10), where viability percentage associated with each concentration is available within related bars.

Based on these observations, MTT analyses were repeated using a narrower concentration range (1–50 µM) with increased sampling density to improve the resolution and reproducibility of the dose–response results. The optimized results have been reported in Figure S8A–F, Section S2.1 (Supplementary Materials). Under these experimental conditions, compounds **1**–**3** exhibited more consistent cytotoxic profiles. In HTLA 230 cells, all three compounds showed considerable cytotoxicity with limited dependence on concentration and treatment duration. In HTLA ER cells, compounds **1** and **2** maintained marked cytotoxic activity, whereas compound **3** showed only modest effects (Figure S8A–F). Since this second set of experiments generated more reproducible and biologically coherent profiles, these data were considered for subsequent analyses. To confirm these assumptions and give a rationale for our choice of what results to present, a study concerning residuals was made inside a one-way ANOVA test for compound **1**, taken as the reference compound. Specifically, two kinds of plot were considered and reported in Figure S9A–D in the Supplementary Materials. Common residuals plots and homoscedasticity plots (Figure S9A,B and Figure S9C,D, respectively) provided similar images. In the images, residuals for the second set of experiments assumed a uniform vertical width, well separated in three groups, each one for a time treatment, against those for the first tests, where the cloud of residuals fanned out or narrowed. Collectively, modelling the first set of results, a heteroscedastic plot was obtained, whereas when modelling the second set of results, homoscedastic plots were revealed. Additionally, the experiments performed within the 1–50 µM range indicated that the maximal cytotoxicity of the compounds was achieved at relatively low concentrations (1–20 µM). Based on these findings, additional experiments with the most active compound **7**, were carried out using further restricted concentration ranges (1–10 µM). The corresponding results have been reported in Figure S10A,B in Section S2.1 (Supplementary Materials), while the optimized dose–response profiles, in the form of dispersion graphs for compounds **1**–**10** have been summarised in Figure 1A–T. Statistical significance has been not repeated on these graphs since it is already reported in the corresponding bars graphs reported in the Supplementary Materials (Figures S7, S8 and S10).

To quantitatively compare the cytotoxic activity of semisynthetic compounds **1**–**7** between each other and with that of the corresponding natural pristine triterpenes **8**–**10**, dose–response data obtained from the viability assay shown in Figure 1 were further analysed to determine IC_50_ values. Cell viability percentage was plotted versus Log_10_-transformed concentrations and then analysed by a non-linear regression method (log(inhibitor) versus normalized response-variable Hill slope) suggested by PRISM GraphPad software 8.0.1. The resulting IC_50_ values at the different times of exposure are reported in Table 2.

#### 2.4.1. Activity Potency Category of Compounds **1**–**10** Related to IC_50_ Values Reported in the Literature

To better define the cytotoxic activity of compounds **1**–**10**, the IC_50_ values obtained in this study were compared with those reported in the literature for natural and semisynthetic triterpenoids tested in NB models. A summary of published IC_50_ commonly used to classify the anticancer activity of triterpenoid-based compounds, is reported in Tables S1 and S2 in the Supplementary Materials (Section S2) [11,54,55,56,57,58,59,60,61,62,63,64]. To our knowledge, no previous study has reported the effects of triterpenes or their derivatives in HTLA 230 or HTLA-ER cell lines. Most available articles report that natural triterpenes such as betulin (BET), betulinic acid (BA), lupeol, etc., often exhibit IC_50_ values in the mid µM range, often above 10 µM in NB cells less aggressive than HTLA [26,62,65]. However, the cytotoxic activity of these molecules appears to be dependent on the specific NB phenotype investigated. In fact, the cell type and time of treatments can strongly affect the anticancer outcomes of identical molecules [4,5]. In this regard, MYCN-amplified HTLA 230 cells are considered among the most aggressive model for metastatic NB [66], and therefore represent a particularly reliable system for testing in vitro novel anticancer candidates. Additional information regarding the relative aggressiveness and anticancer treatment responsiveness of different NB cell models has been provided in Section S2.1.2 [6,66,67,68,69,70,71,72,73,74,75,76,77] of the Supplementary Materials, after Table S2.

Low values of IC_50_ were observed in SK-N-AS cells [26,62,65], while higher IC_50_ values were reported in NB-1 cells [62]. In the present study, BET displayed even higher IC_50_ values (25.9–98.3 µM) in HTLA 230 cells, consistent with their highly aggressive phenotype. Conversely, optimized derivatives and/or heavily derivatized triterpenoids have been reported to achieve markedly improved anticancer activity (IC_50_ values below 10–20 µM) depending on the type of the chemical modification [26,78]. EB25, developed in the year 2015 (IC_50_ < 1 µM), can be considered exceptional and promising as a lead compound, and is worthy of further development [66,78].

#### 2.4.2. Cytotoxic Potency of Compounds **1**–**7** and Their Natural Cores **8**–**10** Against HTLA Cells

In NB studies, compounds displaying IC_50_ values ≤ 10 µM should be considered biologically relevant, whereas slightly higher thresholds may still indicate promising anticancer activity in the MDR model depending on the mechanism of action, tumour aggressiveness, and selectivity towards healthy cells [12]. Natural triterpenes (e.g., oleanane, ursane, and lupane scaffolds) often show IC_50_ values within 5–30 µM in NB cell lines such as IMR-32, SK-N-BE, SHEP [71,79] and SH-SY-5Y [56,78,80,81]. However, substantial variability has been reported in the literature, highlighting the strong influence of both biological and experimental conditions on the cytotoxic effects [82]. Previous investigations demonstrated that semi-synthetic derivatization can markedly increase the anticancer activity of such triterpenes, in some cases leading to IC_50_ values within the 1–10 µM range [83] and, more rarely, below 1 µM [82], when optimized lipophilicity and mitochondrial targeting properties are achieved [26,84]. Nevertheless, studies specifically evaluating semisynthetic triterpenes in highly aggressive HTLA cells [66] are very limited [15,16]. Therefore, the findings of the present study may provide relevant information regarding the therapeutic potential of these compounds, especially on treatment-refractory NB cells. Compound **1** was synthesized by combining the propargyl carbamate on C-28 of BET, reported to inhibit the Akt/ERK1/2 kinase system in NB cells [26], and the TPP^+^ hexanoic ester group at the C-3 position, known for its mitochondria-targeting anticancer effects against HTLA 230 and ER cells [11,40,54]. Structurally, compound **1** could partially resemble the derivatives EB25/1 previously reported by Król et al. [26]. Pristine BET (**8**) demonstrated weak cytotoxicity in HTLA 230 and HTLA ER cells with IC_50_ values ranging from 25.87 µM (72 h) to 98.28 µM (24 h), depending on treatment exposure and cellular type (Table 2). The fold increase in anticancer activity using compound **1**, with respect to its origin (BET), is reported in Table 3, for both cell lines and at all exposure times.

Collectively, the activity of BET was significantly enhanced against both HTLA 230 and HTLA ER. Despite the more remarkable enhancement of BET anticancer effects observed in HTLA 230 cells, compound **1** also maintained strong activity against HTLA ER, suggesting that the combined presence of the propargyl and TPP^+^ moieties may effectively enhance the cytotoxic potential of the parental triterpene scaffold. Moreover, the activity of compound **1** generally increased with treatment duration, particularly in HTLA 230, consistent with a time-dependent antiproliferative effect. Interestingly, despite the highly aggressive phenotype of HTLA 230 cells, compound **1** showed greater potency than the related derivative EB25/1 previously tested in SK-N-AS NB cells [26]. This finding may support the beneficial contribution of the TPP^+^ group in improving anticancer efficacy against highly refractory NB models. Compound **2** corresponds to the C-28 acetylenic carboxylate BET derivative previously reported by Król et al. as EB5, a compound with a stronger anticancer effect than the related derivative EB25/1 on SK-N-AS cells [26]. Here, the same chemical modification markedly improved the anticancer activity of pristine BET in HTLA 230 and ER cells (Table 3). Interestingly, compound **2** showed even greater efficacy on ER cells with respect to HTLA 230, similarly to what happens for pristine BET. In HTLA ER cells, a potent anticancer activity was already observed at 48 h (IC_50_ = 9.95 µM) and further increased after 72 h (IC_50_ = 8.02 µM), corresponding to a remarkable improvement compared to BET (Table 3). Moreover, in both cell populations, the cytotoxic effects of compound **2** showed a time-dependent trend, with increasing activity at longer exposure times. As with compound **2**, compound **3** did not contain the TPP^+^ group, but only the antiproliferative acetylenic triple bond, and was obtained by introducing a propargyl amide on the C-28 carboxyl of the BA *core*. This modification was designed to exploit the biological activity associated with acetylenic moieties, previously reported to interfere with signalling pathways involved in NB cell proliferation and survival [26]. Pristine BA displayed a moderate cytotoxic effect in both cell lines (Table 2), showing a major anticancer effect on ER cells (Table 2). These findings support the drug-refractory nature of HTLA 230 compared to other MYCN-amplified NB cells. In fact, BA has been reported to exhibit IC_50_ values ranging from 2 to 10 µg/mL (4.4–21.9 µM) after 72-h treatments in IMR-32 and SH-EP NB cells, including anti-CD95 and DOX-resistant clones of SH-EP [85]. In the present study, higher IC_50_ values were observed in HTLA 230, confirming a lower sensitivity to BA treatment [66]. Derivatization of BA to compound **3** resulted in a remarkable enhancement of cytotoxic activity in HTLA 230 (Table 3). Compound **3** exhibited a time-dependent increase in the activity, reaching IC_50_ values of 5.47, 1.53, 1.17 µM after 24, 48 and 72 h of treatment, respectively. Notably, compound **3** is one of the two best achievements of this study, exhibiting marked time-dependent killing kinetics on HTLA 230, with potency increasing to borderline sub-micromolar values, especially after 72-h treatments. These results indicate that the introduction of the propargylamide moiety substantially enhances the anticancer properties of the BA scaffold on MYCN-amplified NB cells (Table 3). In contrast, compound **3** displayed a minor cytotoxic activity on HTLA ER and was less effective than the parental BA scaffold (Table 3). This finding suggests that, although highly active on drug-sensitive NB cells, the mechanism of action of compound **3** may not efficiently overcome the drug resistance acquired by HTLA ER cells. Since no studies evaluating the effects of triterpenoids derivatives in HTLA 230 or HTLA ER cells are currently available, the comparison with the literature data was necessarily extended to other NB cells [62]. In this regard, the study of Król et al., investigating acetylenic synthetic derivatives of (BET) in SK-N-AS helped us in such a comparison [26]. Among the compounds described, 28-*O*-propargyloxycarbonylbetulin (EB25/1) is structurally the most comparable to BA-derivative **3**, as both derivatives contain the acetylenic moiety in the form of propargyl groups on the primary hydroxyl in C-28. The SK-N-AS cell line is a hyper diploid NB model with a very aggressive phenotype, originally derived from the bone marrow metastasis of a six-year-old female patient [26,74]. Nevertheless, unlike HTLA 230 cells, they do not harbour MYCN amplification. Consistent with this distinction, SK-N-AS cells underperform HTLA 230 cells, in terms of aggressivity and natural refractory behaviour to common chemotherapeutic agents, as demonstrated by the 10-times lower IC_50_ exerted against BET. In this context, the low-micromolar activity displayed by compound **3** against HTLA 230 is noteworthy. Indeed, despite the differences in cellular models and experimental conditions, compound **3** showed greater potency than the related derivative EB25/1 reported by Król et al. [26]. Despite being very poorly active against HTLA ER, the remarkable efficacy observed in MYCN-amplified HTLA 230 cells, with IC_50_ values as low as 1.17 µM, after 72 h, supports further investigation of this derivative as a promising lead structure for HR NB. Compound **4** was obtained as **3**, by decorating BA. In addition to the insertion of propargyl amine on the carboxyl group in C-28, the TPP^+^ group was inserted as a hexanoic ester in C-3 as in compound **1**. Unfortunately, chemical modification of **9** (BA) to **4** dramatically reduced BA activity on HTLA 230, under all time treatments (Table 3). In contrast, compound **4** showed higher cytotoxicity on HTLA ER (Table 2), but not sufficient to ratain it of further experiments. Compared with BA, a modest increase in the activity was observed, particularly at longer exposure times (Table 3). Interestingly, unlike compound **3**, which was highly active on HTLA 230 but poorly effective on HTLA ER, compound **4** displayed the opposite trend, suggesting that the introduction of the TPP^+^ moiety may alter the cellular determinants responsible for the sensitivity to these derivatives. Since its potency remained lower than that of compounds **1** and **2**, despite the fact that the preferential activity exerted on the resistant cells warrants consideration, in our opinion, further investigations on 4 are not advisable. As observed for several other derivatives, the cytotoxic effect of compound **4** increased with treatment duration, particularly in HTLA ER. Compounds **5** and **6** deriving from the chemical modification of BET showed very similar cytotoxic effects on both NB cell populations, with weak deviations. Both compounds were much more active on HTLA ER cells than HTLA 230, and in both cases, the activity was slightly time dependent. For compound **5**, cytotoxic activity on HTLA ER was approximately higher than that on HTLA 230, by 8.2 (24 h), 7.2 (48 h) and 5.5-fold (72 h). A comparable trend was observed for compound **6**, whose activity on HTLA ER exceeded that on HTLA by 7.3 (24 h), 7.9 (48 h) and 8.7-fold (72 h). This preferential cytotoxic activity on MDR cells is particularly interesting, as overcoming acquired resistance represents one of the main objectives of our study. Collectively, compounds **5** and **6** did not ameliorate the activity of the original triterpenoid (BET) on HTLA 230 (Table 3). Anyway, both derivatives resulted in marked activity on HTLA ER, for which available therapeutic agents fail (Table 2), with the derivatives strongly outperforming their original source (Table 3). Compound **7**, deriving from the chemical modification of UA, emerged as the most promising molecule identified in this study. UA has multi-target pharmacological activities, including antimicrobial and anticancer potential, but its therapeutic potential is limited by poor aqueous solubility and bioavailability, if not opportunely modified. The chemical modifications introduced in compound **7** markedly enhanced the anticancer activity of the parental scaffold, generating a derivative with remarkable potency on drug-sensitive and multidrug-resistant NB cells (Table 2 and Table 3). With respect to the observations for compounds **1**, **2**, **4**, **5** and **6**, compound **7** was slightly more active on HTLA 230, than HTLA ER, and remained the best-performing compound, endowed with potent/exceptional anticancer activity. Based on these very promising results, we believe that now, compound **7** is the best candidate for further investigations on cancer cells, performed in this study, and on healthy cells, which will be carried out in the future. These characteristics also make compound **7** particularly attractive for future preclinical investigations not in the scope of this study. However, compounds **1** and **2** represent very promising multitarget molecules to be developed as new weapons against both NB cell populations, while compound **3** is active on HTLA 230. A classification of the anticancer potency of compounds **1**–**10**, together with additional comparisons with the original triterpenoids is provided in Tables S3 and S4. Section S2.1.3 of the Supplementary Materials provides comparisons of our compounds with previously reported triterpenoids derivatives [3,20,78,80,81,86,87,88,89].

#### 2.4.3. Comparison of the Cytotoxic Activity of Compounds **1**–**10** with That of Etoposide (ETO)

**Figure 2:**
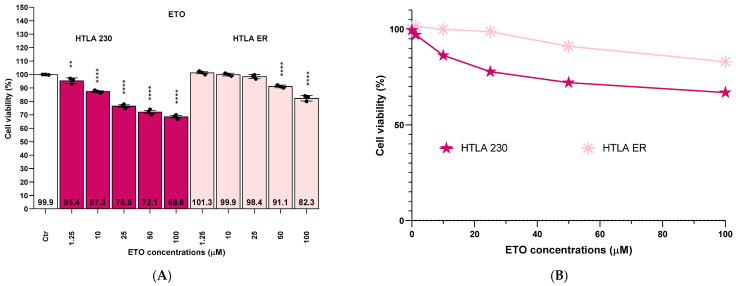
Cell viability of HTLA 230 (strong-pink bars (**A**) and strong-pink line (**B**)) and HTLA ER (soft-pink bars (**A**) and soft-pink line (**B**)) after 24-h exposure to increasing concentrations of ETO (1.25–100 µM). (**A**) shows the bar graph of cell viability (%) vs. increasing ETO concentrations (1.25–100 µM), while (**B**) shows the corresponding dispersion graphs. The viability of untreated cells was assumed as the control (Ctr, white bar (**A**)). Bar graphs summarize quantitative data of the means ± S.D. of four independent experiments (black spheres) run in triplicate. Significance refers exclusively to control (Ctr) and was reported using the * symbol. Specifically, no symbols *p* > 0.05; **** *p* < 0.0001; and ** *p* < 0.01 (one-way ANOVA followed by Dunnett’s multi-comparisons test). Numbers above bars indicate viable cells (%) [3,40].

Considering that a clinically relevant therapy currently applied to treat HR-NB consists of a drug cocktail containing ETO [90,91], the activity of compounds **1**–**10** was compared with that of ETO under the same experimental conditions. Cytotoxicity data for ETO in HTLA 230 and HTLA ER after 24 h of exposure [3,40] are reported in Figure 2.

**Figure 3:**
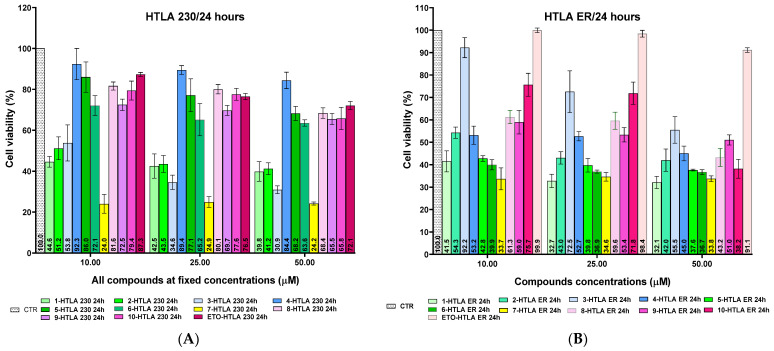
Comparison between HTLA 230 (**A**) and HTLA ER (**B**) cell viability % after 24-h exposure to fixed concentrations (10, 25 and 50 µM) of ETO (red bars for HTLA 230 and pink bar for HTLA ER) and compounds **1**–**10** (bars coloured as in the legend). Specifically, the three dark pink colours indicate natural triterpenoids (BET **8**, BA **9** and UA **10**); the four green colours indicate the triterpenoid derivatives **1**, **2**, **5** and **6** containing BET; the two blue colours indicate compounds **3** and **4** deriving from BA; and finally, yellow bars indicate compound **7** derived from UA. The viability of untreated cells was assumed as the control (100%, punctuated with bars). Bars sum up quantitative data of the means ± S.D. of four independent experiments run in triplicate [3,40].

According to Figure 2, when ETO was administered as a single agent, it displayed limited anticancer activity in HTLA 230 and HTLA ER cells. Even at the highest concentration tested (100 µM), cell viability did not decrease to under 50%, reaching values of 68.6 (HTLA 230) and 82.3% (HTLA ER). These findings are consistent with the intrinsic refractoriness of HTLA 230 cells and with the acquired resistance of HTLA ER. To facilitate comparison among compounds, Figure 3 shows the viability of HTLA 230 (A) and of HTLA ER (B) after exposure to fixed concentrations (10, 25 and 50 µM) of ETO and triterpenoids **1**–**10**.

ETO, at a 50 µM concentration, displayed a limited cytotoxicity (cell viability of 72.1% and 91.1% in HTLA 230 and HTLA ER, respectively; Figure 3). Under the same conditions, all natural and semisynthetic compounds considered in this study, except for compound **4** in HTLA 230 (50 µM), were more active than ETO, regardless of their phenotype (Figure 3). In HTLA 230, natural triterpenes (**8**–**10**) and BET derivatives **5** and **6** shared a similar cytotoxic activity, with 64–68% viable cells even at 50 µM (Figure 3A). Despite this weak activity, they were all slightly better performing than ETO (only 38% of killed cells) by 1.1-fold. On the contrary, compound **4** represented the least active derivative, with 84.4% of cell viability at 50 µM (Figure 3A). Conversely, compounds **1**, **2**, **3** and **7** resulted in a rapid decrease in cell viability reaching values in the range of 24.2–41.2% at 50 µM concentrations and thus outperforming ETO by 1.8–2.9-fold (Figure 3A). Among them, compound **7** was the most cytotoxic, already reducing HTLA 230 cell viability by 55% at 2 µM (Figure 3A). The superiority of the triterpenoid derivatives was even more evident in HTLA ER, where ETO was essentially inactive, with 99.9%, 98.4%, and 91.1% cell viability at 10, 25, and 50 µM, respectively. Under the same conditions, most derivatives reduced cell viability below 50% (Figure 3B). Only natural triterpene **9** and BA derivative **3** never decreased cell viability under 50%, reaching the minimum values of 51% and 55.5% at 50 µM. In contrast, compounds **1**, **5**, **6** and **7** consistently displayed strong cytotoxicity, with viability values ranging between 32% and −44% at 50 µM, thus outperforming ETO (91.1%) by 2.1–2.8-fold (Figure 3B). Notably, compound **7** was capable to kill 67.7% of HTLA ER cell and to reduce their cell viability to 32.2% at only 5 µM. Table 4 summarizes the relative performance of triterpenoids versus ETO (Figure 3B in terms of the ratio between the cell viability (%) of HTLA 230 and HTLA ER exposed to ETO and that of cells exposed to compounds **1**–**10** at 10, 25 and 50 µM concentrations. An additional table (Table S5) in the Supplementary Materials provides the ratio between IC50 (µM) ETO and compounds **1**–**10**, when used to treat HTLA 230 and HTLA ER.

In HTLA 230 (Figure 3A, Table 3), most compounds outperformed ETO, whereas compound **4** remained less active. In HTLA ER, all compounds proved to be superior to ETO, with activity efficacy improved by 1.1–3.0-fold, depending on concentration and derivative. To provide a more quantitative comparison, IC_50_ values for ETO were calculated from the dose–response curves shown in Figure 2B.

**Figure 4:**
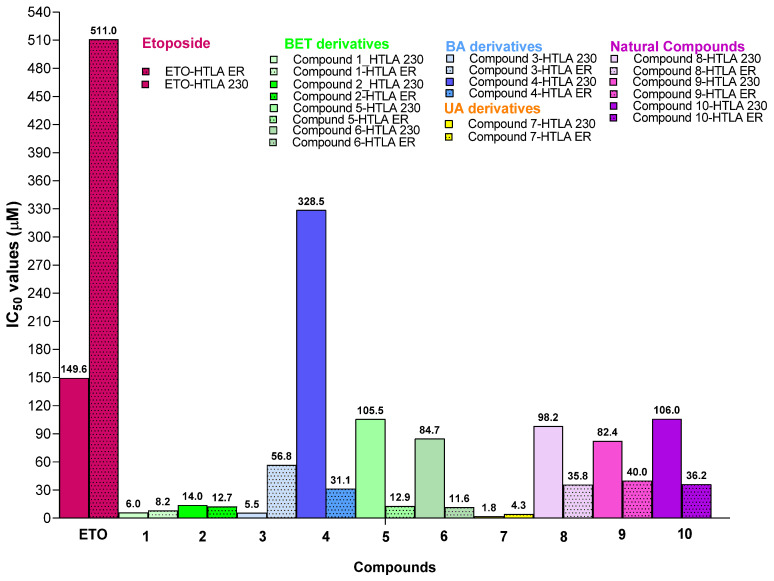
Comparison between the IC_50_ values of compounds **1**–**10** vs. HTLA230 (no patterned bars) and HTLA ER (patterned bars) and those of ETO (red bars), considering treatments of 24 h. Numbers above bars indicate the related IC_50_ values. Green bars with different intensities refer to BET derivatives **1**, **2**, **5** and **6** according to the legend; light-blue bars refer to BA derivatives **3** and **4** according to the legend; and yellow bars refer to UA derivative **7**. Pink to purple bars refer to BET (**8**), BA (**9**) and UA (**10**), according to the legend.

Table S4 and Figure 4 show that ETO had IC_50_ values of 149.6 µM and 511.0 µM in HTLA 230 and HTLA ER cells, respectively. By comparison, natural triterpenoids (**8**–**10**) and newly synthesized **1**–**3** and **5**–**7** displayed lower IC_50_ values, exceeding ETO cytotoxicity by 1.4–32.9-fold in HTLA 230 and by 6.7–65.5-fold in HTLA ER (Figure 4). Only compound **4**, and only on HTLA 230, displayed IC_50_ values higher than that of ETO, while it was superior to ETO by more than 16-fold against HTLA ER. Overall, these in vitro results highlight the remarkable activity of all of these triterpenoids, particularly compounds **1**, **2**, **5**, **6** and **7**, on HTLA 230 and HTLA ER cells. The strong superiority over ETO (by one to two orders of magnitude), especially on HTLA ER, suggests that these semisynthetic triterpenoids, may act through mechanisms that differ, at least in part, from those responsible for the cytotoxic effects of ETO [26].

#### 2.4.4. Additional Biological Experiments

The limited anticancer effects of ETO observed in parental HTLA 230 (IC_50_ = 149.6 µM), further confirm the intrinsic refractoriness of HTLA 230 cells. In this context, compounds capable of exerting potent cytotoxic effects on HTLA 230 remain of considerable interest. For example, compound **3**, although only weakly active on HTLA ER, has a potent anticancer effect on HTLA 230, reducing cell viability by nearly 60% at 5 µM and reaching IC_50_ values as low as 1.17 µM after 72 h of treatment. Nevertheless, from a translational perspective, derivatives capable of maintaining strong activity in both sensitive and MDR NB cells are particularly attractive. Among the compounds investigated, derivatives **1**, **2**, and especially **7** fulfilled this requirement, showing low micromolar IC_50_ values in both cell populations. In detail, after 24 h, compounds **1**, **2** and **7** showed IC_50_ values of 8.23, 12.65 and 7.8 µM in ER cells and 5.99, 13.96 and 4.5 µM in HTLA 230, respectively. The ability of these compounds to exhibit cytotoxic effects at low doses and with short exposure times (24 h) may represent a favourable feature for future investigation and development. Although their selectivity and safety profiles remain to be established, the data suggest that effective biological activity can be achieved at concentrations markedly lower than those of ETO in the same experimental model. Based on its outstanding activity in both HTLA 230 and HTLA ER, compound **7** was selected for further biological characterizations, including clonogenicity assays and functional studies. These experiments were carried out to better define its anticancer activity and to assess its potential as a lead compound for future preclinical investigations.

#### 2.4.5. Effect of Compound **7** on Clonogenic Potential of HTLA 230 and HTLA ER

**Figure 5:**
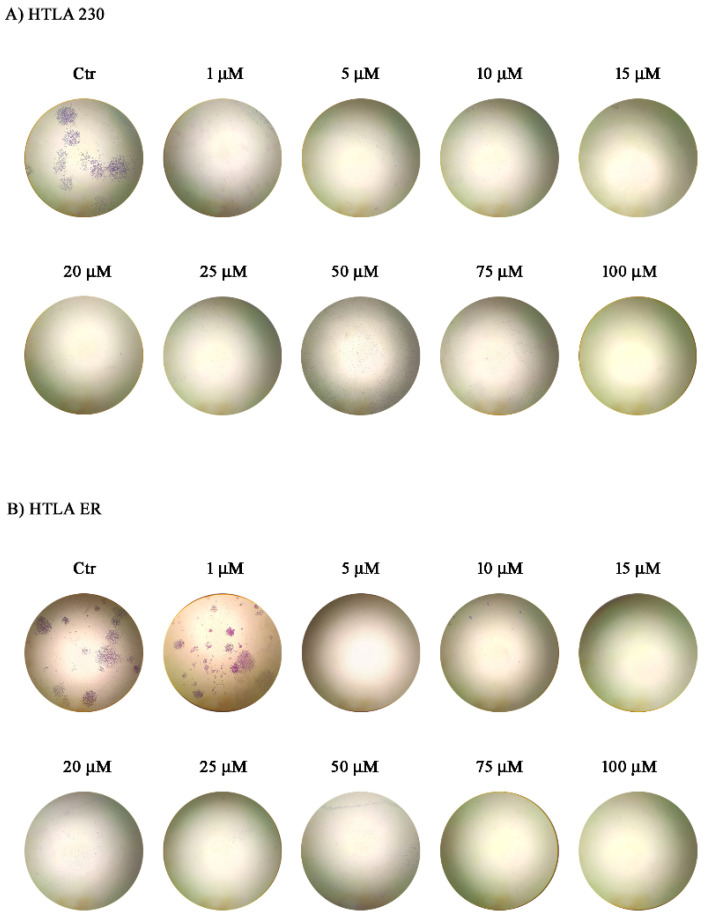
Effect of compound **7** on the clonogenic potential of HTLA 230 and HTLA ER. Representative clonogenic assays performed on HTLA 230 (**A**) and HTLA ER (**B**). Both cell populations were seeded in 6-well plates and then treated for 24 h with compound **7** at increasing concentrations (1–100 µM). After 15 days, colonies were stained with crystal violet and imaged using an optical microscope.

To determine whether the cytotoxic effects of compound **7** can be translated into a long-term suppression of cancer cell reproductive capacity, clonogenic assays were performed in HTLA 230 and HTLA ER. Since to count colonies (more than 50 for clonogenicity), a manual count is currently the most used method, because it is simple, inexpensive, and does not require specialized software, colony counts were carried out manually. Colony formation assays provide a valid evaluation of the anticancer efficacy, as they evaluate the ability of surviving cells to retain unlimited proliferative potential and to generate macroscopic colonies after drug exposure. Compound **7** strongly impaired the clonogenic capacity of both NB cell populations (Figure 5). In HTLA 230, a complete inhibition of colony formation was already observed at 1 µM, indicating that even very low doses of the compound were sufficient to abolish the ability of surviving cells to undergo sustained proliferation. A similarly dramatic effect was observed in HTLA ER, although a complete suppression of colony formation was obtained at 5 µM.

These findings are particularly interesting when evaluated together with MTT results, which demonstrated a marked dose-dependent cytotoxic activity of compound **7**. Importantly, clonogenic assays revealed an even more profound biological effect, as residual surviving cells were unable to re-establish proliferative colonies, indicating a complete loss of tumour-repopulating capacity in both NB cell populations [92]. This sustained impairment of proliferative potential is consistent with growing evidence suggesting that mitochondria-targeted triterpenoid derivatives can exert durable antiproliferative effects through disruption of mitochondrial function, bioenergetic collapse, and activation of cell death pathways [93,94]. Particularly, TPP^+^-conjugated triterpenoids have been reported to accumulate preferentially in mitochondria, thereby enhancing anticancer activity and retaining efficacy even in drug-resistant cancer models.

Taken together, the complete suppression of clonogenic growth achieved by compound **7** at low micromolar concentrations, suggest this derivative as a particularly promising candidate for further development against both drug-sensitive and MDR NB cells. However, while clonogenic assays provide robust evidence of long-term loss of tumour-repopulating capacity, they do not disclose the molecular mechanisms underlying these effects.

In this context, the strongly positive ζ-potential (>+60 mV) observed for compound **7** suggests a high propensity for interaction with negatively charged cellular membranes and supports efficient cellular uptake and mitochondrial accumulation, consistent with its cationic lipophilic scaffold. Accordingly, MTT assays revealed a pronounced dose-dependent reduction of cellular metabolic activity, indicating an early impairment of mitochondrial redox function. Therefore, to elucidate the molecular basis of the irreversible loss of clonogenicity, key regulators of survival signalling, stemness maintenance, and apoptotic execution—including Akt/p-Akt, BMI1, and PARP processing—were assessed. Anyway, further experiments, not in the scope of this study, such as direct tests for caspase activity or annexin staining, are necessary to confirm deductions deriving from results from experiments carried out in this study.

#### 2.4.6. Functional Characterization of Compound **7** in MDR NB Cells

**Figure 6:**
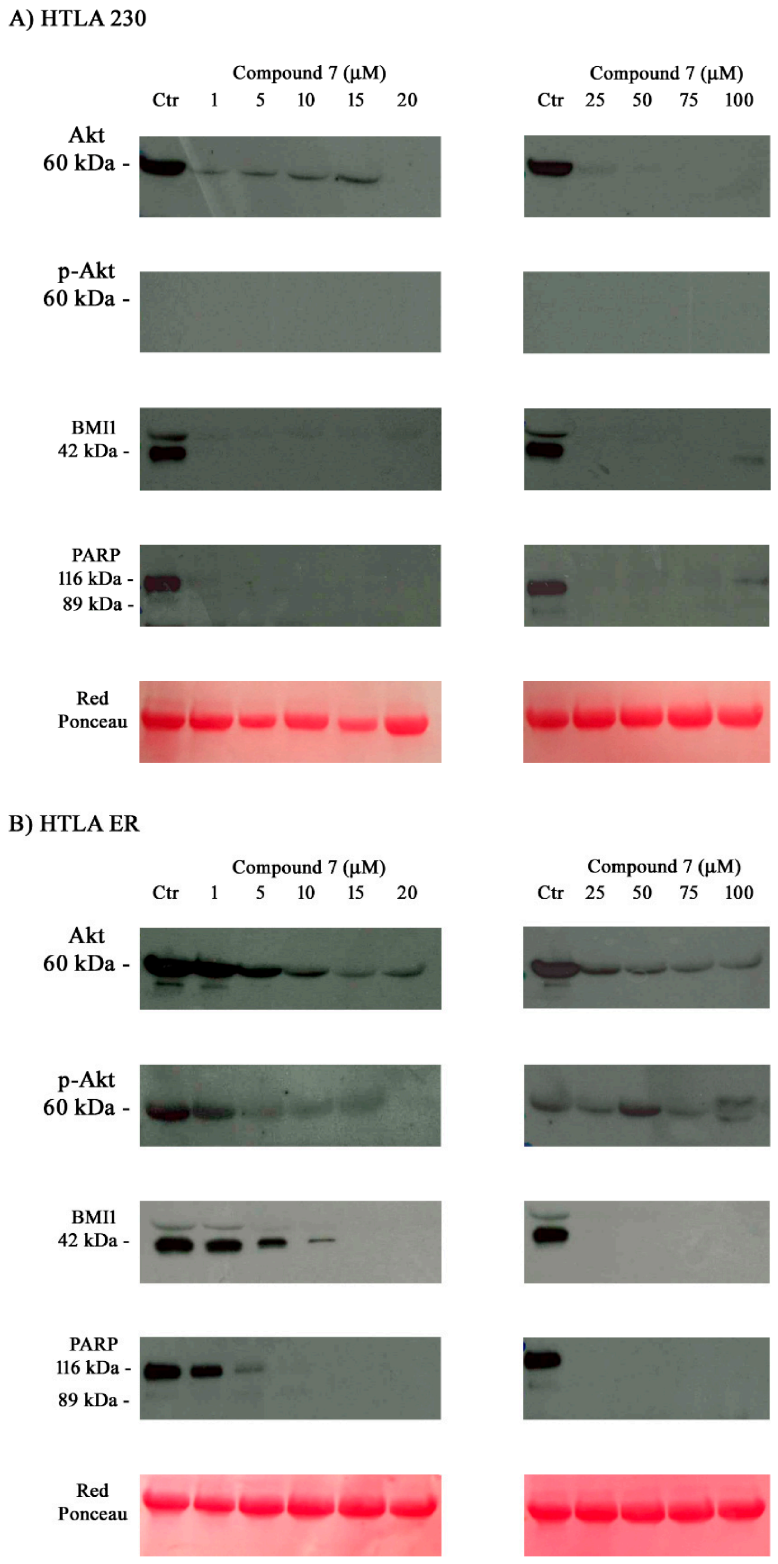
Modulation of Akt signalling, BMI1 expression, and PARP levels by compound **7** in drug-sensitive and MDR NB cells. Representative immunoblot analysis of total Akt, phosphorylated Akt (*p*-Akt), BMI1, and PARP in HTLA 230 (**A**) and HTLA ER (**B**) treated with increasing concentrations of compound **7**. Red Ponceau staining was used to verify equal protein loading and transfer efficiency. Molecular weights (kDa) are indicated alongside each blot. Blots are representative of three independent experiments with comparable outcomes.

Compound **7** induced a concentration-dependent reduction of total Akt protein levels in HTLA 230 and HTLA ER (Figure 6). Phosphorylated Akt (p-Akt) was detected only in HTLA ER, where it progressively decreased following treatment with compound **7** (Figure 6). These findings suggest biological differences of Akt pathway activation in NB cell populations showing that compound **7** interferes with Akt-associated signalling, particularly in MDR cells. Akt signalling downregulation is consistent with its established role as a central driver of NB aggressiveness, metabolic adaptation, and therapeutic resistance [95,96].

Furthermore, in HTLA ER, compound **7** induced a marked dose-dependent reduction of BMI1 expression, while, in contrast, in HTLA 230, BMI1 levels became barely detectable following the treatment (Figure 6). This finding is consistent with the demonstrated role of the miR-15/16–BMI1 regulatory axis in NB progression and cellular self-renewal capacity [97], and suggests that compound **7** may interfere with molecular programs associated with cancer survival and multidrug resistance.

In addition, PARP analysis revealed that in HTLA 230, full-length PARP was readily detectable in untreated cells but became undetectable following exposure to compound **7**, whereas no cleaved PARP fragment was observed. In HTLA ER, a progressive concentration-dependent reduction of full-length PARP expression was detected, and in this case, PARP cleavage products were not detectable. The lack of the catalytic PARP fragment argues against the activation of a canonical caspase-dependent apoptotic program under these experimental conditions. Instead, these data integrated with the marked inhibition of mitochondrial metabolic activity, observed in MTT assays, the complete suppression of clonogenic growth, and the concomitant downregulation of Akt and BMI1, suggest a mechanism of action consisting of a severe loss of cellular homeostasis and bioenergetic collapse, ultimately leading to irreversible cell death rather than to an apoptotic response [98,99]. Such an interpretation is further supported by the mitochondria-targeting ability of compound **7** and by its highly positive ζ-potential, which helps its cell membrane interaction, its intracellular accumulation, and the mitochondrial dysfunction.

Taken together, compound **7** emerged as a highly effective multitarget agent capable of counteracting key features of NB aggressiveness and multidrug resistance. Its remarkable activity at low micromolar concentrations, combined with the complete suppression of clonogenic potential and the disruption of Akt/BMI1-associated survival networks, highlights this derivative as an attractive lead compound for further preclinical studies. Anyway, we are aware that the complete disappearance of the total PARP pool without detection of degradation products may indicate total protein lysis, autophagy, or even a technical artifact during the transfer or detection of low-molecular-weight fractions, rather than a specific bioenergetic collapse. Thus, we recognize that, the statement about non-apoptotic death could need additional rigorous confirmation by conducting direct tests for caspase activity or annexin staining, which were not in the scope of this study.

### 2.5. Structure–Activity Relationship Considerations

As reported above, according to Król et al., the acetylenic triple bond bound to C-28 of BET is capable of exerting moderate to potent anticancer activity on SK-N-AS, but not MYCN-amplified NB cells, via Akt kinase system inhibition, depending on its structure [26]. When present as acetylene ester (compound EB5, **2** in this study), a potent sub-micromolar anticancer activity was observed after 96-h treatments (IC_50_ = 0.62 µM), while when present as propargylamine, as in compound EB25/1, the cytotoxic potency was more moderate (IC_50_ = 18.77 µM). Concerning the semisynthetic triterpenoids in this study, except for compounds **5** and **6** not bearing the triple bond, thus not working according to Król et al., and **2** which contained the same group as EB5 [26], suggesting the same high activity, all other triterpenes contained the propargylamine group of EB25/1 and, except for **3**,the bond in C-28 with TPP^+^ group, regardless of their different *cores*, suggesting anticancer potency inferior to EB5 and **2**. Anyway, the possible additional presence of the amphiphilic alkyl TPP^+^ cation strongly affected the superficial charge and the overall anticancer potency of the compounds, preferentially redirecting it against MDR HTLA ER cells, in most cases. In these muted NB cells, the inhibition of the Akt system reported by Król et [26], probably more difficult to reach and inhibit, assumed less importance than an effective membrane adhesion and disruptor effect, favouring the entrance of molecules into cells, which was necessary to maintain considerable anticancer potency, sustained by two mechanisms. In fact, compounds not bearing the alkyl TPP^+^ group, but only the Akt inhibitor ones, such as BET derivative **2** and BA derivative **3**, were equally active (**2**, IC_50_ = 8.11 vs. 8.02 µM, 72 h) or more active (**3**, IC_50_ = 1.17 vs. 47.76 µM, 72 h) against drug-sensitive HTLA 230, where the inhibition of kinases was easier, and occurred despite a weak primary adhesion, supported by moderate ζ-p (+28.5 and +29.3 mV). The different activities of **2** and **3** could depend on the different *cores*, as BA is more active than BET against HTLA 230, and less active against ER. On the contrary, BET derivatives **5** and **6**, not bearing acetylenic moieties, were not active against HTLA 230, where killing mechanisms depended mainly on blocking the kinase system, but exerted good activity against HTLA ER (**5**, IC_50_ = 6.38, **6** 7.48 µM, 72 h), where adhesion, membrane impairment, cellular uptake and mitochondria targeting, promoted by high ζ-*p* (+83.1 and +50.4 mV), assumed more importance than kinase inhibition for maintaining anticancer potency. BET and UA derivatives **1** and **7**, bearing both the propargylamine and TPP^+^ group, displayed potent activity against both HTLA phenotypes, strongly supported by the possibility of strong adhesions promoted by the high ζ-*p* values (+56.0 and +62.8 mV), with the anticancer potency of **7** against both HTLA 230 and ER being exceptional for triterpene derivatives (IC_50_ = 0.58 and 2.90 µM, 72 h, respectively). The lower activity of **1** with respect to **7**, against HTLA 230 and ER, could depend on the different chemical insertions of propargylamine, with the propargylamide group being a better choice than the propargyl carbamate one. Curiously, BA derivative **4**, as equally decorated as UA derivative **7**, was completely inactive against HTLA 230 cells, and despite demonstrated good activity against HTLA ER cells, higher than that of EB25/1 by Król et al. in longer treatments, and against non-MDR cells, it displayed anticancer activity significantly lower than that of all other triterpene derivatives bearing the same TPP^+^ group against such phenotypes, probably due to a minor ζ-*p* and the large dimensions of the particles. Collectively, despite the introduction of the TPP^+^ group on C-3 of BA increasing the positive ζ-p of **3** from +29.3 to +45.4 mV, promoting its stronger adhesion, membrane disruption effects, cellular uptake and mitochondria targeting, thus leading to IC_50_ values lower by 3-times on ER cells, it was detrimental against HTLA 230, annihilating the inhibitory effect of the acetylene group seen for **3**, thus causing a 173-times improvement of IC_50_ values. This could suggest that for the activity of BA derivatives against HTLA 230, it is important that the hydroxyl in C-3 remains free.

## 3. Material and Methods

### 3.1. Compounds ***1***–***10***

The source of natural compounds **8**–**10** and the synthetic procedure to synthesize their derivatives **1**–**7**, as well as methods followed to carry out their complete characterization, are detailed in a previous paper [23].

### 3.2. Dynamic Light Scattering Analysis (DLS)

The mean diameter (Z-_AVE_) and polydispersity index (PDI) of amphiphilic compounds **1** and **4**–**7**, and zeta potential (ζ-p) of compounds **1**–**7**, were measured at 25 °C, in water, using a Malvern Nano ZS90 light scattering apparatus (Malvern Instruments Ltd., Worcestershire, UK) at a scattering angle of 90° as previously described [100]. Concentrations of compounds for both Z-_AVE_ determinations and ζ-p measures have been expressed in kcps values and reported in Table 1 in Section 2. The apparent equivalent hydrodynamic radii of sample particles were calculated using the Stokes–Einstein equation, by instrument. Z-_AVE_ results by number (%) were presented and discussed. For both Z-_AVE_ (nm) and ζ-p (mV) determinations, aliquots of compound dispersions at a given kcps were withdrawn, having a count rate (kcps) sufficient for the analysis. The results from these experiments were expressed as mean ± SD of three measurements made of several runs for each one.

### 3.3. Cytotoxicity Experiments Using Compounds ***1***–***10*** on Drug-Sensitive HTLA 230 and MDR HTLA ER Cells

#### 3.3.1. Cell Lines and Culture Conditions

MYCN-amplified HTLA 230 human stage-IV NB cells were kindly provided by G. Gaslini Institute (Genoa, Italy), for research purposes. HTLA ER cells were generated as previously reported [11]. All cell lines were maintained in RPMI 1640 medium (Euroclone Spa, Pavia, Italy) supplemented with 10% foetal bovine serum (FBS, Euroclone Spa, Pavia, Italy), 1% L-glutamine (Euroclone Spa, Pavia, Italy), and 1% penicillin/streptomycin (Euroclone Spa, Pavia, Italy) and grown in standard conditions (37 °C humidified incubator with 5% CO_2_).

#### 3.3.2. Treatments

HTLA 230 and HTLA ER cells, were treated for 24, 48 and 72 h with increasing concentrations (1–100.0 µM) of compounds **1**–**10**. Subsequently, different concentration ranges of 1–50 and 1–10 µM, were tested using compounds **1**–**3** and **7**, for optimizing results. The stock solutions of these compounds were prepared in 40,000-fold diluted DMSO, and preliminary experiments demonstrated that the final DMSO concentrations did not change any of the cell responses analysed. Cell cultures were carefully monitored before and during the experiments to ensure optimal cell density. Notably, samples were discarded if the cell confluence reached >90%.

#### 3.3.3. Cell Viability Assay

Cell viability was determined by using the CellTiter 96^®^ AQueous One Solution Cell Proliferation Assay (Promega, Madison, WI, USA), as previously described [101,102]. Briefly, cells (10,000 cells/well) were seeded into 96-well plates (Corning Incorporated, Corning, NY, USA) and then treated. Next, cells were incubated with CellTiter, and the absorbance at 490 nm was recorded using a microplate reader (EL-808, BIO-TEK Instruments Inc., Winooski, VT, USA). The cell survival rate, expressed as cell viability percentage (%), was evaluated based on the experimental outputs of treated groups vs. the untreated groups (CTR) and was calculated as follows:

CV (%) = (OD_TC_ − OD_B_)/(OD_UTC_ − OD_B_) × 100% (1)

where CV means cell viability; OD, optical density; TC, treated cells; B, blank; and UTC, untreated cells.

Related bar graphs, reporting cell viability (%) vs. increasing concentrations of **1**–**10** were provided by GraphPad Prism 8.0.1 Software (GraphPad Software, Boston, MA, USA). The same software was used to calculate IC_50_ values, by using a non-linear model. Particularly, the bar graphs were converted into the correspondent dispersion graphs. Then, upon conversion of µM concentrations (variable on x axis) in Log_10_ (x), and using a non-linear model which considered the Log_10_ (**1**–**4** concentrations) vs. the normalized response (variable or not variable slope), the IC_50_ values of all compounds for both cell populations at 24, 48 and 72 h of treatment were derived.

#### 3.3.4. Clonogenicity Assay

HTLA 230 and HTLA ER cells (150 per well) were seeded in twenty-four-well plates (Corning) and treated with compound **7** (1–100 µM) for 48 h. Subsequently, the medium was changed, and the cells were maintained in drug-free medium for two weeks. Cells were then fixed with methanol (MeOH) and stained with crystal violet (CV), 0.5% in water with 50% MeOH. Specifically, clonogenic assays were performed in six independent replicates for each experimental condition. Approximately 150 cells were seeded per well, yielding about 45–50 colonies in untreated control cultures after two weeks, thus confirming an adequate plating efficiency under our experimental conditions.

Colony-count statistical analysis was not performed because treatment with compound **7** resulted in the complete suppression of colony formation at all tested concentrations in HTLA-230 cells and at all concentrations except the lowest one in HTLA-ER cells. Under these conditions, quantitative statistical comparison of colony numbers would not have provided additional biological information beyond the complete loss of clonogenic potential, which is clearly illustrated in Figure 5.

Anyway, a manual counting of colonies present at the lowest concentration was preliminarily made, since to count colonies (more the 50 for clonogenicity), a manual count is currently the most used method because it is simple, inexpensive, and does not require specialized software. More than 50 cells were counted in this experimental condition, and the images were acquired with a Nikon Coolpix L22 camera (Nikon Corporation, Tokyo, Japan).

#### 3.3.5. Western Blot Analysis

Western blot was carried out according to standard methods [103], using the following antibodies: polyclonal rabbit anti-Akt, anti-phospho-Akt, anti-BMI1, anti-PARP and anti-rabbit secondary antibodies coupled with horseradish-peroxidase (Cell Signalling Technology Inc., Danvers, MA, USA). Signals were detected using an ECL chemiluminescence system detection kit (Pierce™ ECL Western Blotting Substrate, Thermo Fisher Scientific). After chemiluminescent detection, images were acquired using a digital imaging system (Bio-Rad ChemiDoc MP Gel Imaging System, Bio-Rad Laboratories (CAT#: STEM-E-2051-LGZ, New York, NY, USA)).

Three independent biological replicates were analysed for experimental conditions. For each replicate, cells were cultured, treated, and lysed separately to ensure biological variability was captured. Normalization to control condition was set to 1 (fold change vs. control). Densitometric analysis was performed using ImageJ/Fiji (version 1.54, National Institutes of Health, Bethesda, MD, USA).

### 3.4. Statistical Analysis

Statistical significance was obtained using GraphPad PRISM software 8.0.1 by the analysis of variance (two-way ANOVA) corrected for multiple comparisons using statistical Dunnet hypothesis testing. The statistical difference of multiple comparisons was reported for each bar in bar graphs and each point in dispersion graphs, when not redundant, for each concentration and for each compound tested using symbols. Symbols used have been specified in this text and in the Supplementary Materials. Specifically, *p* values adjusted for multiple comparisons were reported for each comparison. No symbol was reported when *p* > 0.5. One symbol was used for *p* < 0.1, two, *p* < 0.01, three, *p* < 0.001 and four, *p* < 0.0001.

## 4. Conclusions

In conclusion, this work provides evidence that rationally designed semisynthetic triterpenoids can effectively target MDR HR NB cells through biological effects that differ from those typically observed for conventional chemotherapeutics. Particularly, compound **7** emerged as a lead candidate, displaying durable anticancer activity associated with complete loss of clonogenic potential, marked impairment of cellular homeostasis and modulation of cellular survival-associated proteins. The absence of detectable PARP cleavage, together with the strong inhibition of metabolic activity and irreversible suppression of clonogenic growth, suggests that compound **7** may induce a non-classical cell death response rather than a canonical caspase-dependent apoptotic pathway. Anyway, further investigations are suggested. Although mitochondrial dysfunction was not directly demonstrated, the physicochemical properties of compound **7** and the observed biological effects are consistent with a mechanism involving mitochondria-associated cellular stress. These findings support triterpenoid-based amphiphilic derivatives as attractive platforms for the development of next-generation treatments for therapy-refractory NB.

## Figures and Tables

**Table 1:** Average hydrodynamic diameter (Z_AVE_, nm) and PDI obtained by records acquired by number (%) at the highest kcps (second and third column); average zeta-potential (ζ-p, mV) obtained by records acquired at the highest kcps (fourth column).

Sample	Z_AVE_ (nm), kcps	PDI	ζ-p (mV), kcps
**1**	253.4 ± 23.5, 120.1	0.403	56.0 ± 5.7, 681.5
**2**	N.D.	N.D.	28.5 ± 4.5, 68.8
**3**	N.D.	N.D.	29.3 ± 4.8, 684.7
**4**	386.4 ± 21.1, 4.4	0.704	45.4 ± 7.63, 93.8
**5**	448.0 ± 101.6, 45.3	1.000	83.1 ± 7.4, 54.3
**6**	276.0 ± 42.5, 4.0	0.625	50.4 ± 7.5, 47.6
**7**	240.0 ± 35.5, 97.2	0.253	62.8 ± 6.1, 438.1

N.D. = Not determined.

**Table 2:** In vitro IC_50_ (µM) of pristine compounds **8**, **9**, and **10** and those (µM) of their chemically modified derivatives **1–7**. Data in the table are the quantitative means ± S.D. of four independent experiments run in triplicate, as specified in the caption of Figure 1.

Compound	HTLA 230			HTLA ER		
	24 h	48 h	72 h	24 h	48 h	72 h
**1 (8)**	**5.99 ± 2.50**	**4.42 ± 1.59**	**3.29 ± 1.34**	**8.23 ± 2.11**	**8.07 ± 2.08**	**6.71 ± 2.20**
**2 (8)**	**13.96 ± 3.59**	**10.70 ± 2.34**	**8.11 ± 1.53**	**12.65 ± 3.85**	**9.95 ± 3.55**	**8.02 ± 2.62**
**3 (9)**	**5.47 ± 2.79**	**1.53 ± 0.83**	**1.17 ± 0.42**	56.79 ± 10.64	56.48 ± 17.86	47.76 ± 12.46
**4 (9)**	328.5 ± 66.5	394.3 ± 92.8	202.9 ± 53.0	31.13 ± 13.90	23.49 ± 10.3	16.05 ± 7.40
**5 (8)**	105.50 ± V.W.	68.87 ± V.W.	35.12 ± V.W.	**12.89 ± 6.37**	**9.51 ± 4.45**	**6.38 ± 2.99**
**6 (8)**	84.67 ± 26.15	75.80 ± 22.10	65.25 ± 18.50	**11.60 ± 6.32**	**9.55 ± 5.05**	**7.48 ± 3.25**
**7 (10)**	**1.75 ± 0.26**	**1.38 ± 0.18**	**0.58 ± 0.19**	**4.30 ± 0.45**	**3.39 ± 0.30**	**2.90 ± 0.27**
**8 (BET)**	98.28 ± 17.77	79.54 ± 20.47	25.87 ± 7.76	35.79 ± 11.91	29.97 ± 9.48	22.71 ± 7.19
**9 (BA)**	82.44 ± 23.76	65.22 ± 18.37	33.06 ± 12.92	39.99 ± 15.98	26.98 ± 12.62	17.06 ± 7.76
**10 (UA)**	106.00 ± 18.31	46.61 ± 5.11	14.68 ± 7.34	37.17 ± 6.83	31.91 ± 5.45	25.49 ± 5.90

First column: in bold black, original triterpenoids: BET = betulin (**8**), BA = betulinic acid (**9**), and UA = ursolic acid (**10**); in bold green, BET (**8**) triterpenoid derivatives: **1**, **2**, **5** and **6**; and in bold red, BA (**9**) triterpenoid derivatives: **3** and **4**. Compound **7** (bold light blue) is a UA (**10**) derivative. Compound concentrations were 1–50 µM for **1**, **2** and **3**, 1–10 µM for **7**, and 1–100 µM for all other compounds. V.W. = very wide. Data in bold represents the more promising results. Data in bold in row 7 represents the lowest IC_50_ value observed in this study. Reported IC_50_ values are the quantitative means ± S.D. of four independent experiments run in triplicate, as provided by PRISM software already cited, which intrinsically consider a 95% confidence interval.

**Table 3:** Fold changes with administration to both HTLA 230 and HTLA ER NB cells versus related original cores at 24, 48 and 72 h of treatment.

Compound	HTLA 230			HTLA ER		
	Fold-Change vs. BET	Fold-Change vs. BA	Fold-Change vs. UA	Fold-Change vs. BET	Fold-Change vs. BA	Fold-Change vs. UA
**1 ** 24 h	16.40735	**N.A.**	**N.A.**	4.348724	**N.A.**	**N.A.**
**1 ** 48 h	5.905444	**N.A.**	**N.A.**	3.713755	**N.A.**	**N.A.**
**1** 72 h	15.0713	**N.A.**	**N.A.**	3.384501	**N.A.**	**N.A.**
**2 ** 24 h	5.905444	**N.A.**	**N.A.**	3.161265	**N.A.**	**N.A.**
**2 ** 48 h	6.095327	**N.A.**	**N.A.**	2.711558	**N.A.**	**N.A.**
**2** 72 h	4.076449	**N.A.**	**N.A.**	2.127182	**N.A.**	**N.A.**
**3 ** 24 h	**N.A.**	15.0713	**N.A.**	**N.A.**	**0.704173**	**N.A.**
**3 ** 48 h	**N.A.**	42.62745	**N.A.**	**N.A.**	**0.477691**	**N.A.**
**3** 72 h	**N.A.**	28.25641	**N.A.**	**N.A.**	**0.357203**	**N.A.**
**4 ** 24 h	**N.A.**	**0.322679**	**N.A.**	**N.A.**	1.194025	**N.A.**
**4 ** 48 h	**N.A.**	**0.118209**	**N.A.**	**N.A.**	1.35845	**N.A.**
**4** 72 h	**N.A.**	**0.072351**	**N.A.**	**N.A.**	1.588162	**N.A.**
**5 ** 24 h	**0.931564**	**N.A.**	**N.A.**	2.776571	**N.A.**	**N.A.**
**5 ** 48 h	1.15493	**N.A.**	**N.A.**	3.15142	**N.A.**	**N.A.**
**5** 72 h	**0.736617**	**N.A.**	**N.A.**	3.559561	**N.A.**	**N.A.**
**6 ** 24 h	**0.973662**	**N.A.**	**N.A.**	3.447414	**N.A.**	**N.A.**
**6 ** 48 h	**0.860422**	**N.A.**	**N.A.**	2.825131	**N.A.**	**N.A.**
**6** 72 h	**0.506667**	**N.A.**	**N.A.**	2.280749	**N.A.**	**N.A.**
**7 ** 24 h	**N.A.**	**N.A.**	60.57143	**N.A.**	**N.A.**	8.644186
**7 ** 48 h	**N.A.**	**N.A.**	33.77536	**N.A.**	**N.A.**	9.412979
**7** 72 h	**N.A.**	**N.A.**	25.31034	**N.A.**	**N.A.**	8.789655

In green, BET derivatives; in red, BA derivatives; in yellow UA derivatives; N.A. = not available; in bold, unfavourable changes.

**Table 4:** Ratios between cell viability (%) of HTLA 230 and HTLA ER exposed to ETO and that of cells exposed to compounds **1–10**. Numbers > 1 indicate that ETO was surpassed by our compounds in terms of cytotoxic effects.

	HTLA 230			HTLA ER		
Concentrations	10 µM	25 µM	50 µM	10 µM	25 µM	50 µM
**Compounds**						
**1**	1.957356	1.800919	1.809884	2.408043	3.005605	2.839187
**2**	1.706342	1.757724	1.749557	1.840173	2.286489	2.167525
**3**	1.622198	2.212228	2.331315	1.083689	1.35644	1.641908
**4**	0.945829	0.855705	0.854265	1.877820	1.867173	2.024444
**5**	1.014930	0.991965	1.0565	2.336302	2.475081	2.422696
**6**	1.211427	1.173618	1.133915	2.501397	2.664072	2.481157
**7**	3.637500	3.072272	2.980999	2.964392	2.843213	2.698418
**8**	1.069319	0.955463	1.053432	1.630510	1.650038	2.107994
**9**	1.204787	1.098018	1.100677	1.694683	1.844414	1.785416
**10**	1.099096	0.986177	1.095374	1.320704	1.369842	2.386844

In bold black, compounds compared to ETO; in black, data for compounds which killed more cells than ETO; in red, data for compounds which left alive minimally more cells than ETO; and in blue, data for HTLA ER cells. Numbers > 1 indicate that ETO was outperformed by triterpenes in terms of cell viability reached at that concentration.

## Data Availability

The original contributions presented in this study are included in the article/Supplementary Materials. Further inquiries can be directed to the corresponding authors.

## Supplementary Materials

The following supporting information can be downloaded at https://www.mdpi.com/article/10.3390/ijms27156563/s1.
