# Supplementary material for: Amphiphilic Semisynthetic Triterpenoids Impair Survival Pathways and Suppress Clonogenic Growth in Multidrug-Resistant High-Risk Neuroblastoma
Source: Int J Mol Sci. 2026 Jul 23;27(15):6563. doi: 10.3390/ijms27156563 (PMC13466524; doi:10.3390/ijms27156563)
Supplement: Supplementary file 1 [file ijms-27-06563-s001.zip › ijms-4427463-supplementary.pdf]

Supplementary Materials

Amphiphilic Semisynthetic Triterpenoids Impair Survival Pathways and Suppress Clonogenic Growth in Multidrug-Resistant High-Risk Neuroblastoma

Silvana Alfei [1,*], Cinzia Domenicotti [2,3,*], Sara Tirendi [2,4], Elaheh Khaledizadeh [2], Dafni Graikioti [5], Constantinos M. Athanassopoulos [5], Guendalina Zuccari [1,6], Caterina Reggio [6], and Barbara Marengo [2,3]

[1] Department of Pharmacy, University of Genoa, Viale Cembrano, 16148 Genoa, Italy; guendalina.zuccari@unige.it
[2] Department of Experimental Medicine (DIMES), University of Genova, Via Alberti L.B., 16132 Genoa, Italy; sara.tirendi@edu.unige.it (S.T.); elaheh.khaledizadeh@edu.unige.it (E.K.); barbara.marengo@unige.it (B.M.)
[3] AOM-IRCCS Ospedale Policlinico San Martino, 16132 Genoa, Italy
[4] Inter-University Center for the Promotion of the 3Rs Principles in Teaching & Research (Centro 3R), 56122 Pisa, Italy
[5] Department of Chemistry, University of Patras, University Campus Rio Achaias, 26504 Rio, Greece; dafnigraikioti@upnet.gr (D.G.); kath@upatras.gr (C.M.A.)
[6] Laboratory of Experimental Therapies in Oncology, IRCCS Istituto Giannina Gaslini, Via G. Gaslini 5, 16147 Genoa, Italy; caterinareggio@gaslini.org
* Correspondence: alfei@difar.unige.it (S.A.); cinzia.domenicotti@unige.it (C.D.); Tel.: +39-010-355-2296 (S.A.)

Section S1: DLS Analyses

S1.1. ZAVE (nm) Measurements

Results

			Size (d.n...	% Number:	St Dev (d.n...
**Z-Average (d.nm):**	386,4	**Peak 1:**	63,32	100,0	21,12
**PdI:**	0,704	**Peak 2:**	0,000	0,0	0,000
**Intercept:**	0,794	**Peak 3:**	0,000	0,0	0,000

Result quality Refer to quality report

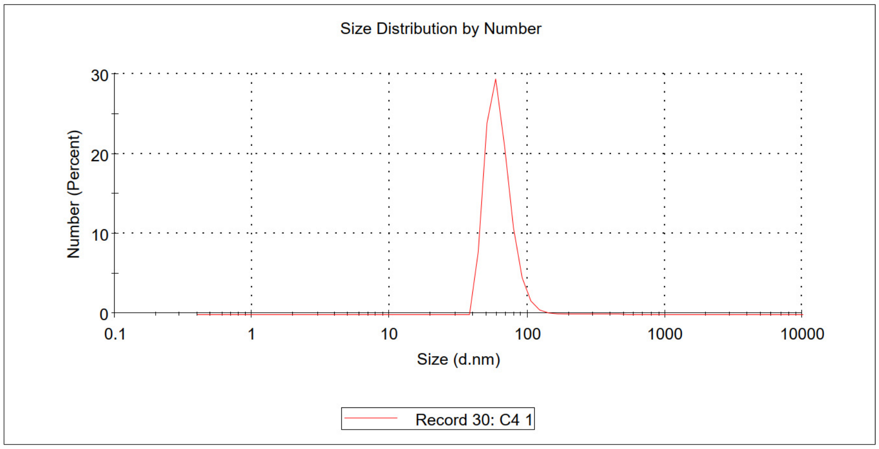

Figure S1. Dimensional distribution (ZAVE, nm) and PDI of compound **4**.

Results

			Size (d.n...	% Number:	St Dev (d.n...
Z-Average (d.nm):	448,0	Peak 1:	153,1	99,9	101,6
PdI:	1,000	Peak 2:	4380	0,1	951,1
Intercept:	0,918	Peak 3:	0,000	0,0	0,000

Result quality Refer to quality report

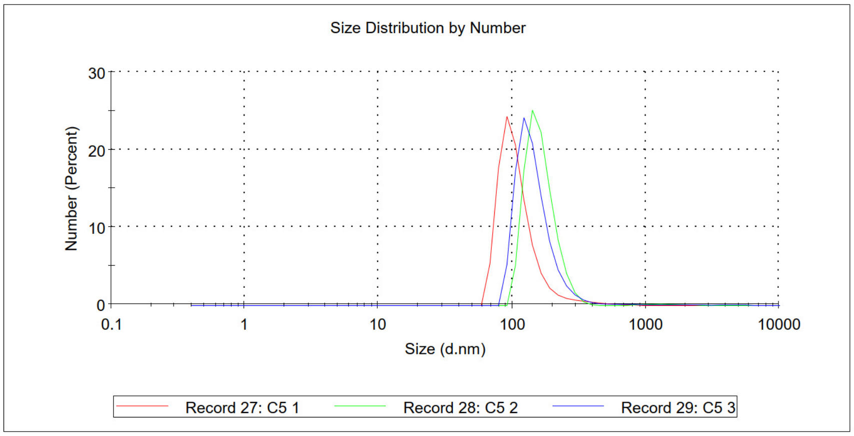

Figure S2. Dimensional distribution (ZAVE, nm) and PDI of compound **5**.

Results

			Size (d.n...	% Number:	St Dev (d.n...
Z-Average (d.nm):	276,2	Peak 1:	144,7	99,7	42,53
PdI:	0,625	Peak 2:	992,5	0,3	326,6
Intercept:	0,728	Peak 3:	4787	0,0	891,9

Result quality Refer to quality report

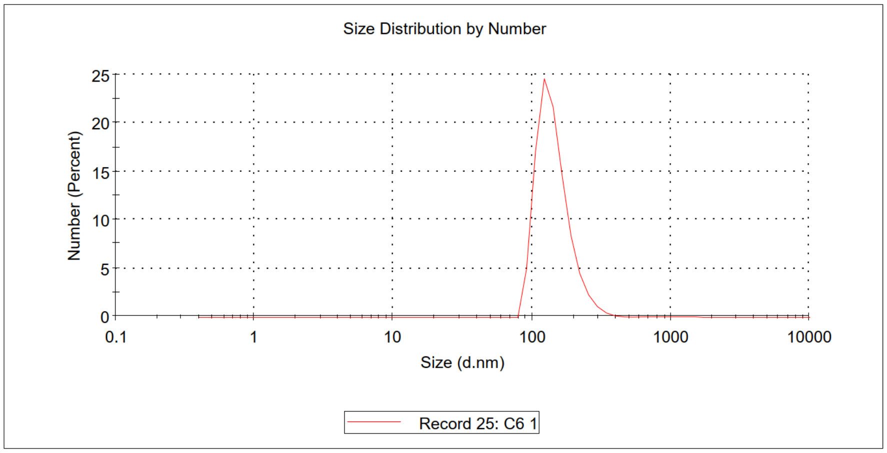

Figure S3. Dimensional distribution (ZAVE, nm) and PDI of compound **6**.

S1.2. ζ-potential (ζ-p, mV) Measurements

Results

			Mean (mV)	Area (%)	St Dev (mV)
**Zeta Potential (mV):**	28,5	**Peak 1:**	28,3	97,9	4,48
**Zeta Deviation (mV):**	4,80	**Peak 2:**	43,7	2,1	1,87
**Conductivity (mS/cm):**	0,0216	**Peak 3:**	0,00	0,0	0,00
**Result quality**	Good				

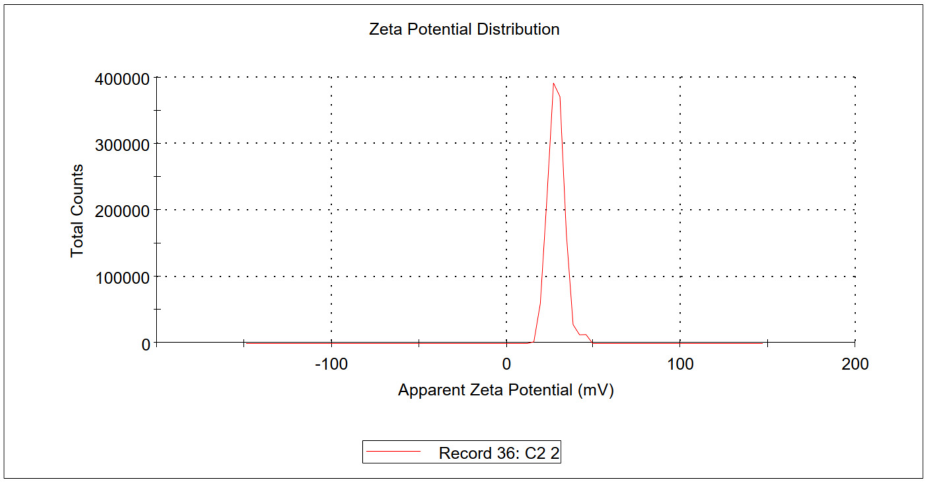

Figure S4. ζ potential (ζ p) distribution (mV) of compound **2**.

Results

			Mean (mV)	Area (%)	St Dev (mV)
**Zeta Potential (mV):**	50,4	**Peak 1:**	50,4	100,0	7,48
**Zeta Deviation (mV):**	7,48	**Peak 2:**	0,00	0,0	0,00
**Conductivity (mS/cm):**	0,0902	**Peak 3:**	0,00	0,0	0,00
**Result quality**	Good				

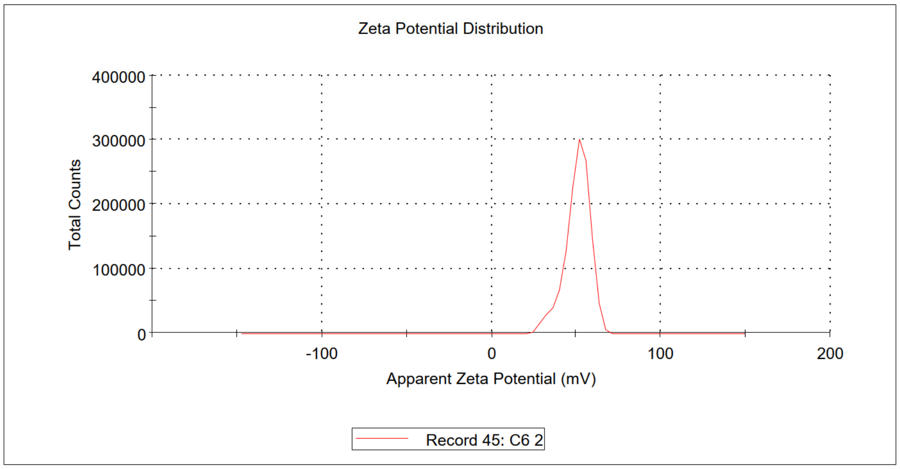

Figure S5. ζ potential (ζ p) distribution (mV) of compound **6**.

Results

			Mean (mV)	Area (%)	St Dev (mV)
Zeta Potential (mV):	62,8	Peak 1:	62,8	100,0	6,08
Zeta Deviation (mV):	6,08	Peak 2:	0,00	0,0	0,00
Conductivity (mS/cm):	0,0445	Peak 3:	0,00	0,0	0,00
Result quality	Good				

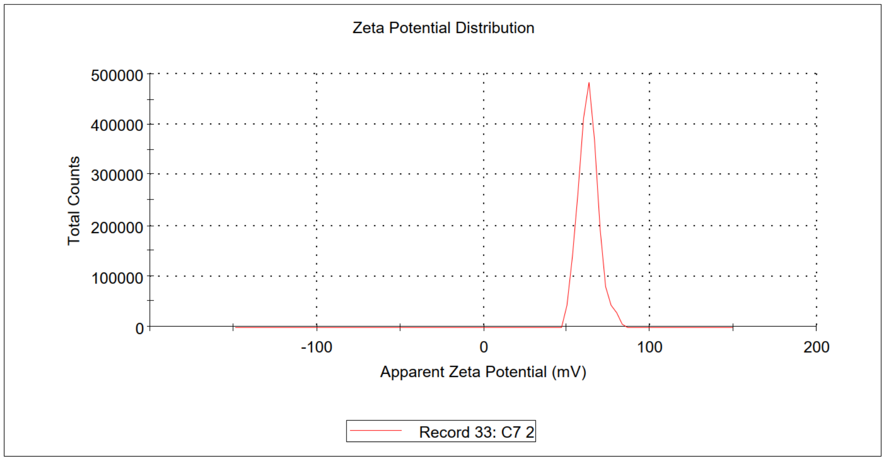

Figure S6. ζ potential (ζ p) distribution (mV) of compound **7**.

Section S2.

S2.1. Cytotoxicity Experiments Against Metastatic HR-NB HTLA Cell Populations

Explorative 24-, 48- and 72-hs experiments were carried out to optimize their conditions against etoposide sensitive and multidrug resistant (MDR) high-risk neuroblastoma (HR-NB), MYCN-amplified HTLA cells (HTLA 230 and ER), using compounds **1-7** (Scheme 1, main text), in the concentration range 1-100 µM. Natural triterpenoids betulin (**8**, BET), betulinic acid (**9**, BA) and ursolic acid (**10**, UA), *cores* of **1-7**, were investigated in the same conditions. Results have been presented as bar graphs of cell viability *vs*. compounds concentrations (Figure S7A-T).

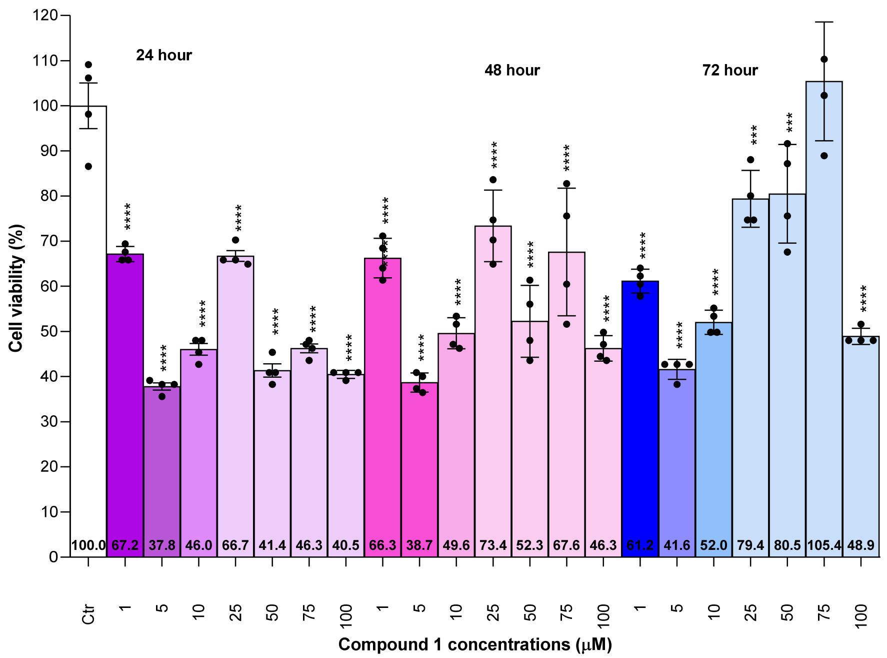

24 hour
48 hour
72 hour
Cell viability (%)
0 10 20 30 40 50 60 70 80 90 100 110 120
100.0 67.2 37.8 46.0 66.7 41.4 46.3 40.5 66.3 38.7 49.6 73.4 52.3 67.6 46.3 61.2 41.6 52.0 79.4 80.5 105.4 48.9
Ctr 1 5 10 25 50 75 100 1 5 10 25 50 75 100 1 5 10 25 50 75 100
Compound 1 concentrations (μM)

(A)

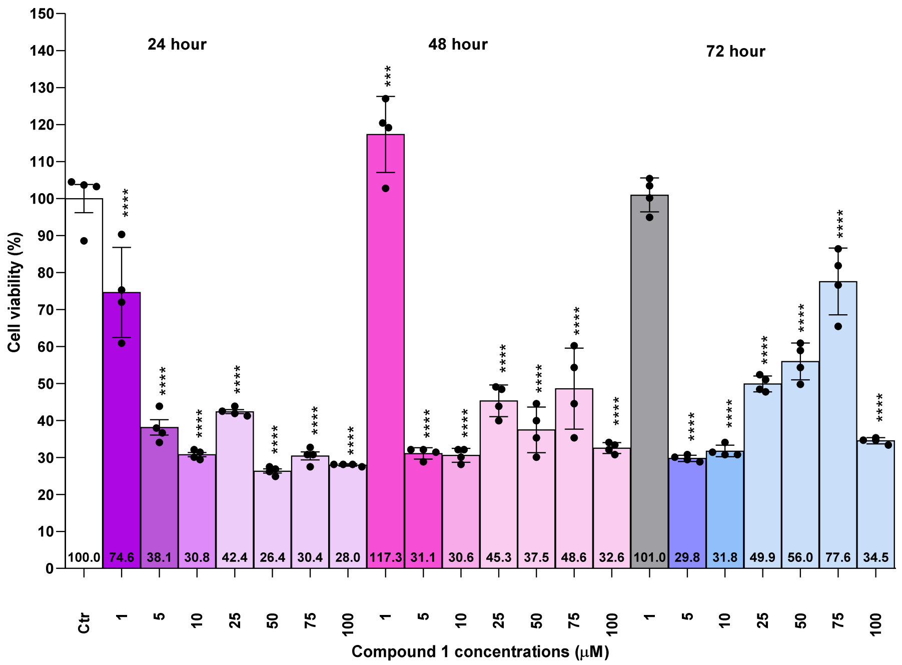

24 hour
48 hour
72 hour
Cell viability (%)
0 10 20 30 40 50 60 70 80 90 100 110 120 130 140 150
100.0 74.6 38.1 30.8 42.4 26.4 30.4 28.0 117.3 31.1 30.6 45.3 37.5 48.6 32.6 101.0 29.8 31.8 49.9 56.0 77.6 34.5
Ctr 1 5 10 25 50 75 100 1 5 10 25 50 75 100 1 5 10 25 50 75 100
Compound 1 concentrations (μM)

(B)

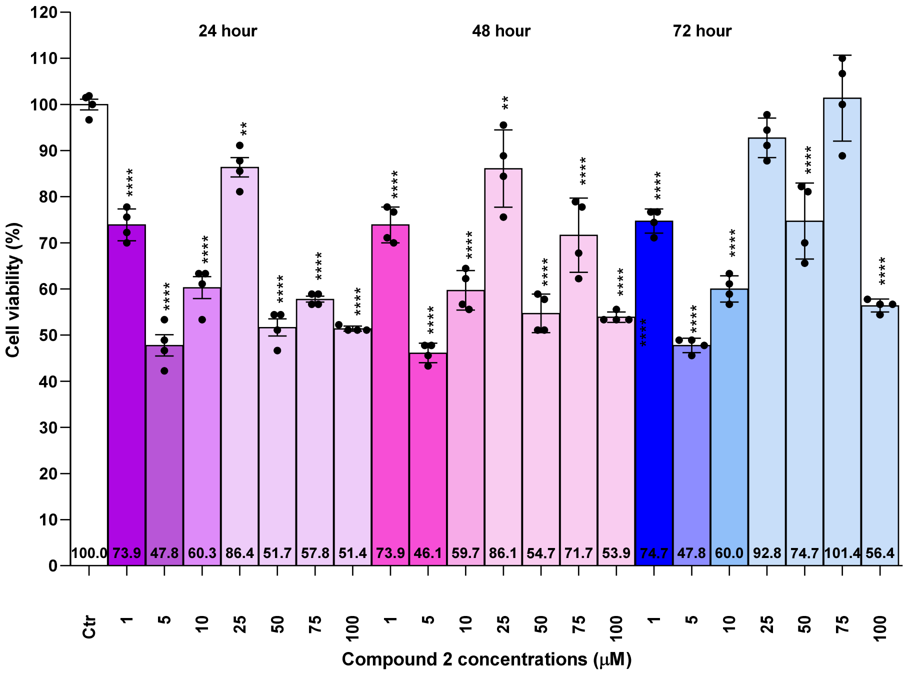
24 hour
48 hour
72 hour
Cell viability (%)
Compound 2 concentrations (μM)
Ctr
100.0
73.9
47.8
60.3
86.4
51.7
57.8
51.4
73.9
46.1
59.7
86.1
54.7
71.7
53.9
74.7
47.8
60.0
92.8
74.7
101.4
56.4

(C)

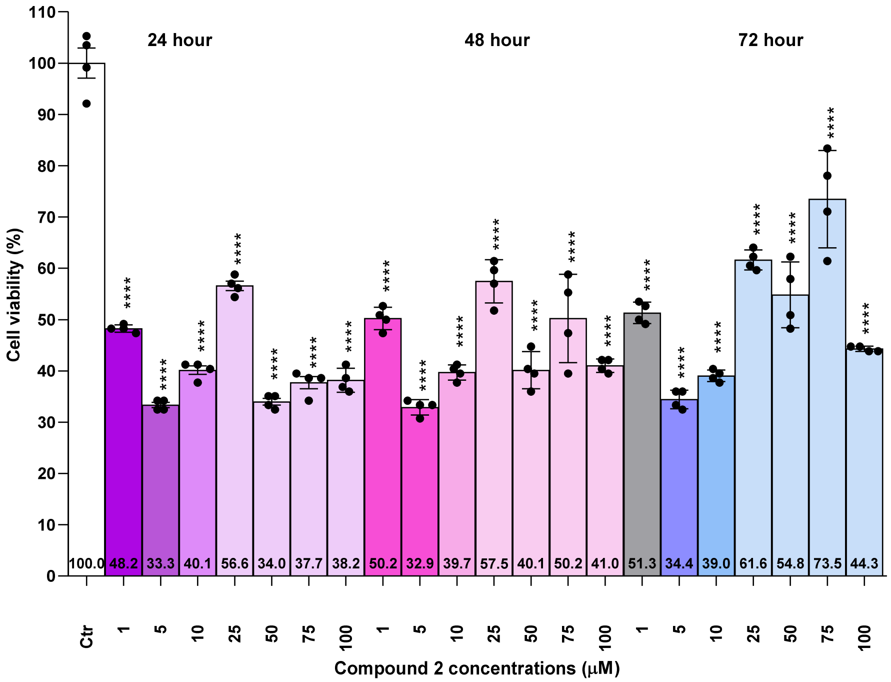
24 hour
48 hour
72 hour
Cell viability (%)
Compound 2 concentrations (μM)
Ctr
100.0
48.2
33.3
40.1
56.6
34.0
37.7
38.2
50.2
32.9
39.7
57.5
40.1
50.2
41.0
51.3
34.4
39.0
61.6
54.8
73.5
44.3

(D)

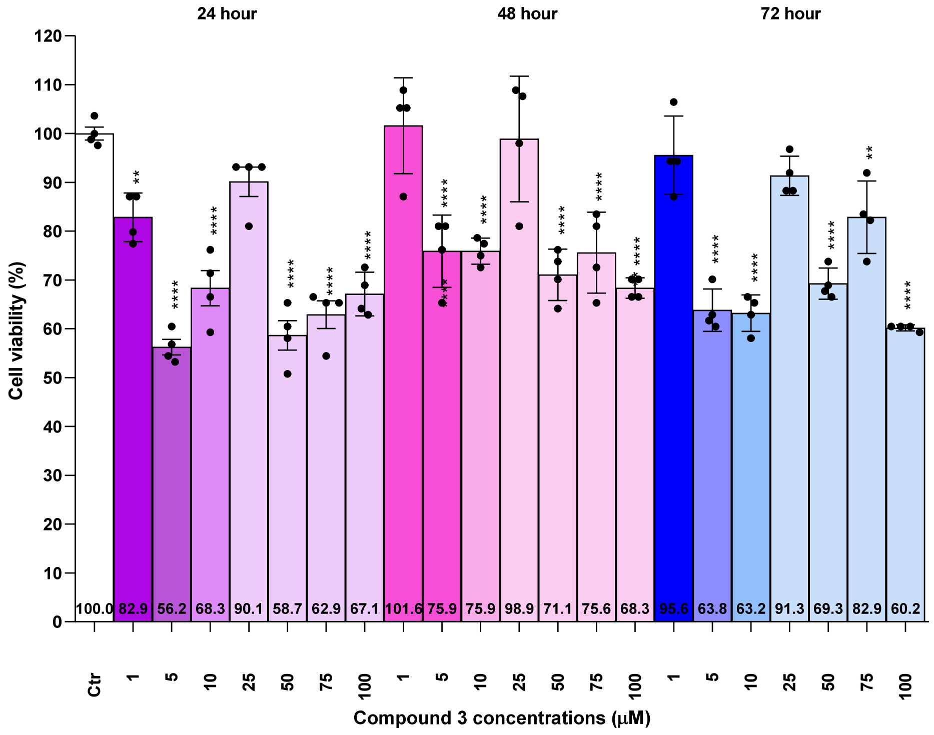

24 hour
48 hour
72 hour
Cell viability (%)
Compound 3 concentrations (μM)

(E)

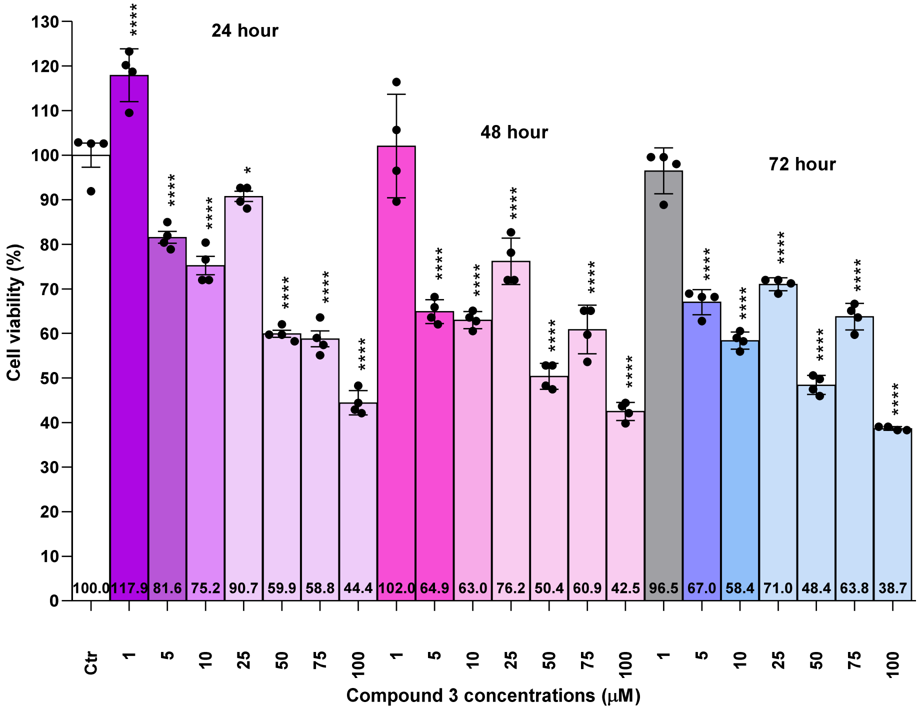

24 hour
48 hour
72 hour
Cell viability (%)
Compound 3 concentrations (μM)

(F)

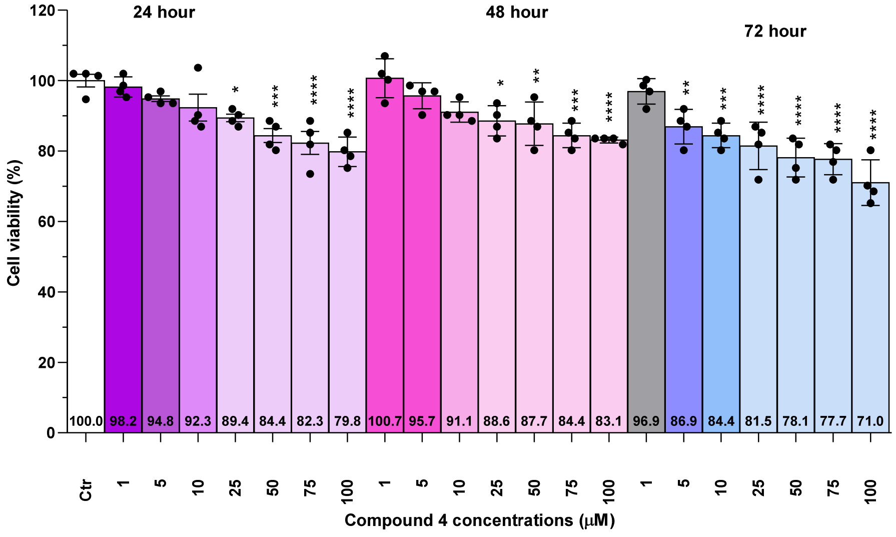
24 hour
48 hour
72 hour
Cell viability (%)
120
100
80
60
40
20
0
100.0 98.2 94.8 92.3 89.4 84.4 82.3 79.8 100.7 95.7 91.1 88.6 87.7 84.4 83.1 96.9 86.9 84.4 81.5 78.1 77.7 71.0
Ctr 1 5 10 25 50 75 100 1 5 10 25 50 75 100 1 5 10 25 50 75 100
Compound 4 concentrations (μM)

(G)

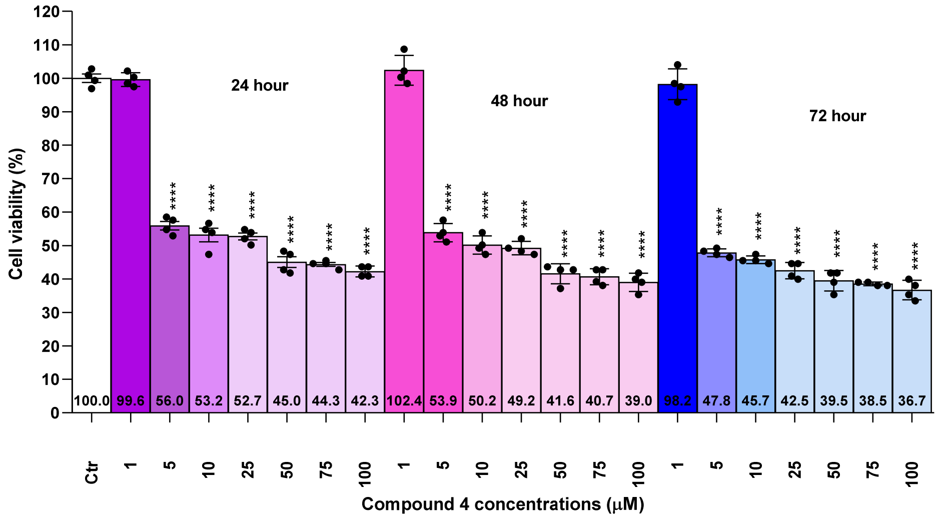
24 hour
48 hour
72 hour
Cell viability (%)
120
110
100
90
80
70
60
50
40
30
20
10
0
100.0 99.6 56.0 53.2 52.7 45.0 44.3 42.3 102.4 53.9 50.2 49.2 41.6 40.7 39.0 98.2 47.8 45.7 42.5 39.5 38.5 36.7
Ctr 1 5 10 25 50 75 100 1 5 10 25 50 75 100 1 5 10 25 50 75 100
Compound 4 concentrations (μM)

(H)

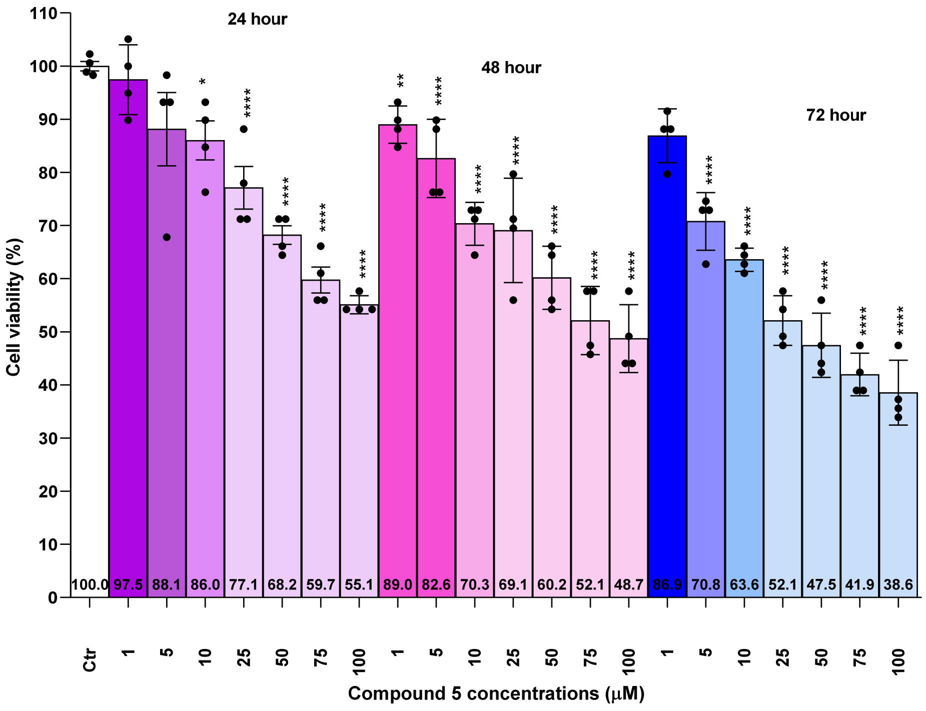

24 hour
48 hour
72 hour
Cell viability (%)
Compound 5 concentrations (μM)
Ctr
1
5
10
25
50
75
100
100.0
97.5
88.1
86.0
77.1
68.2
59.7
55.1
89.0
82.6
70.3
69.1
60.2
52.1
48.7
86.9
70.8
63.6
52.1
47.5
41.9
38.6

(I)

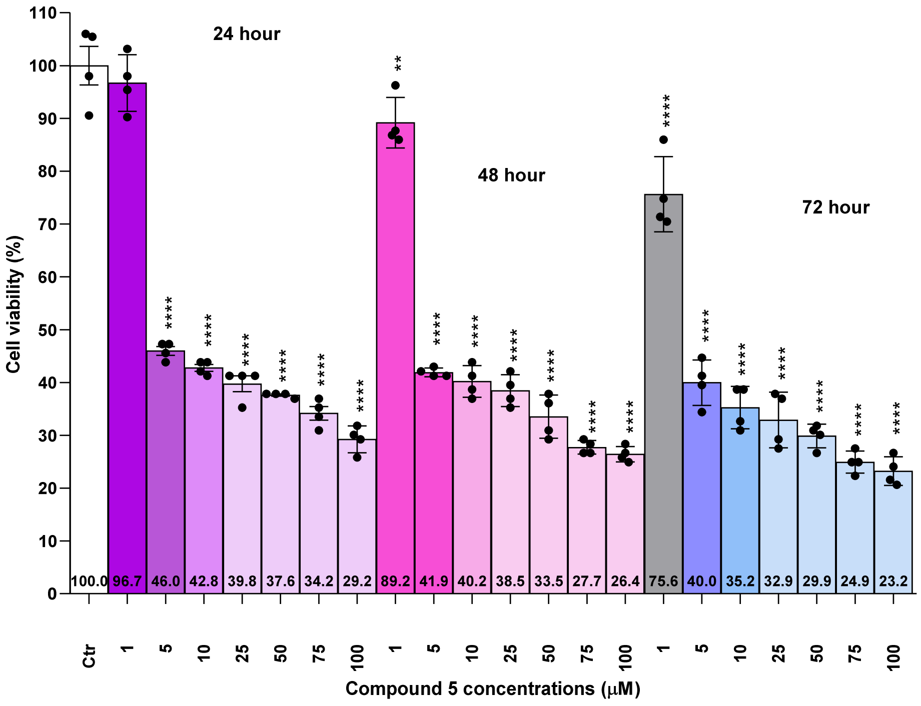

24 hour
48 hour
72 hour
Cell viability (%)
Compound 5 concentrations (μM)
Ctr
1
5
10
25
50
75
100
100.0
96.7
46.0
42.8
39.8
37.6
34.2
29.2
89.2
41.9
40.2
38.5
33.5
27.7
26.4
75.6
40.0
35.2
32.9
29.9
24.9
23.2

(J)

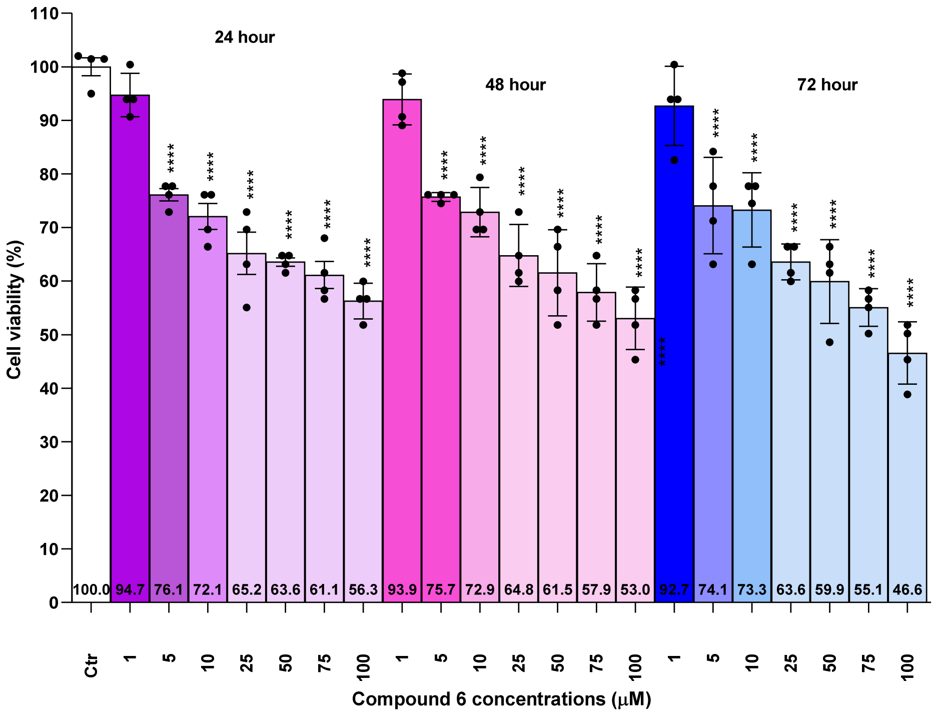

24 hour
48 hour
72 hour
Cell viability (%)
Compound 6 concentrations (μM)

(K)

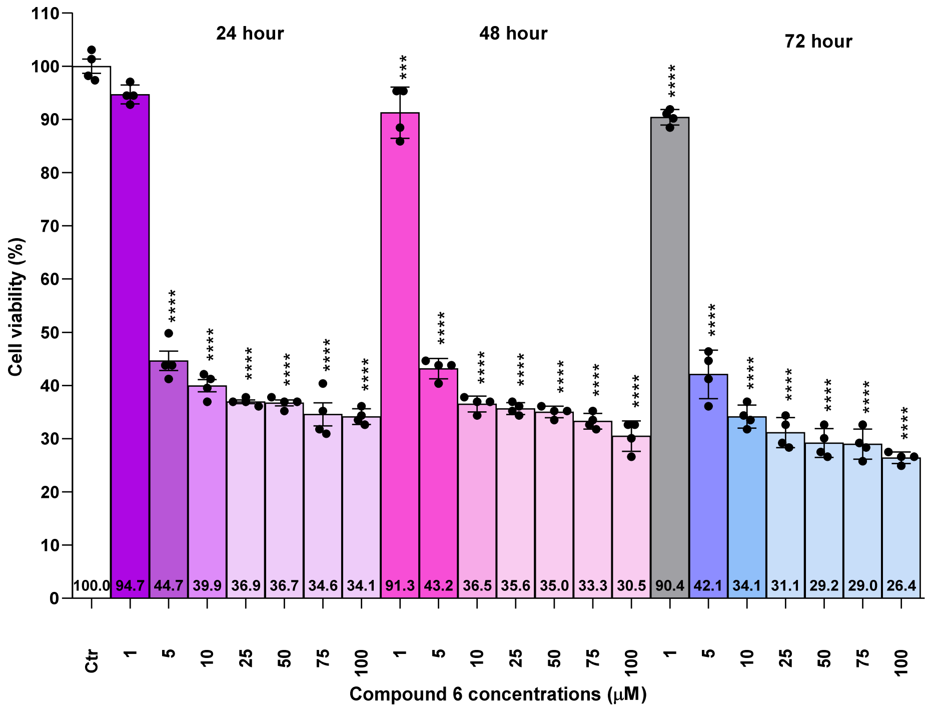

24 hour
48 hour
72 hour
Cell viability (%)
Compound 6 concentrations (μM)

(L)

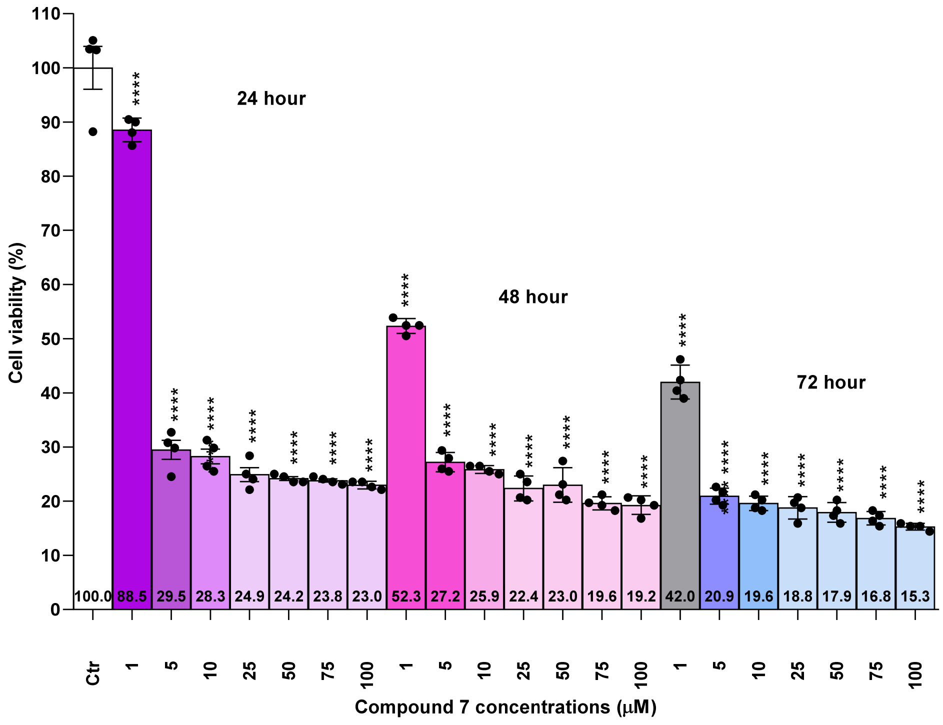

Cell viability (%)
24 hour
48 hour
72 hour
Compound 7 concentrations (μM)

(M)

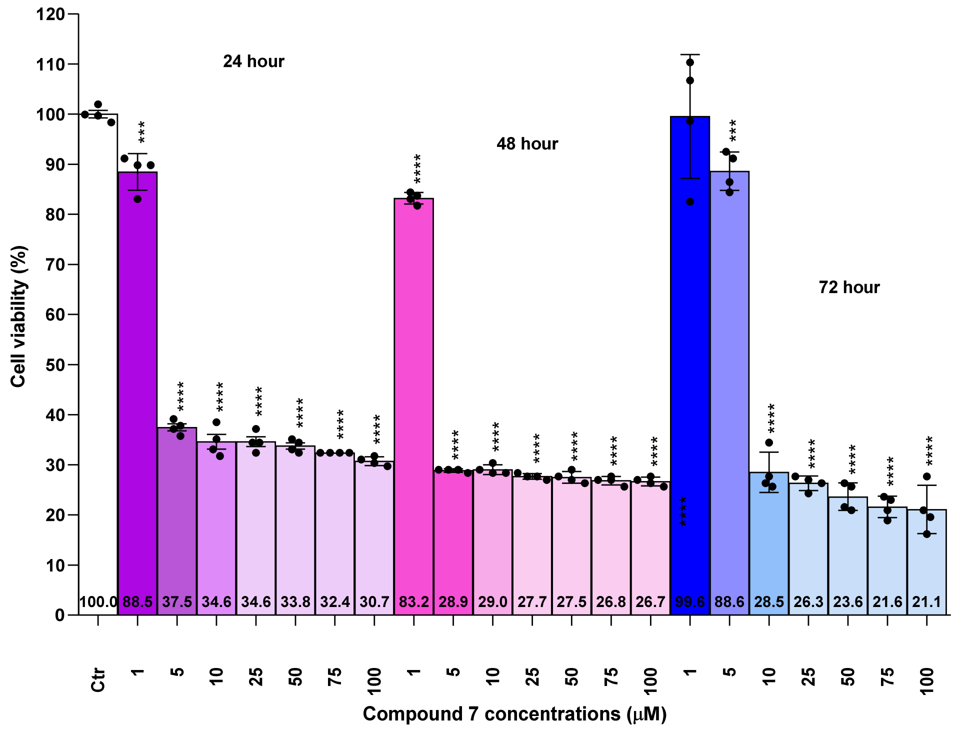

Cell viability (%)
24 hour
48 hour
72 hour
Compound 7 concentrations (μM)

(N)

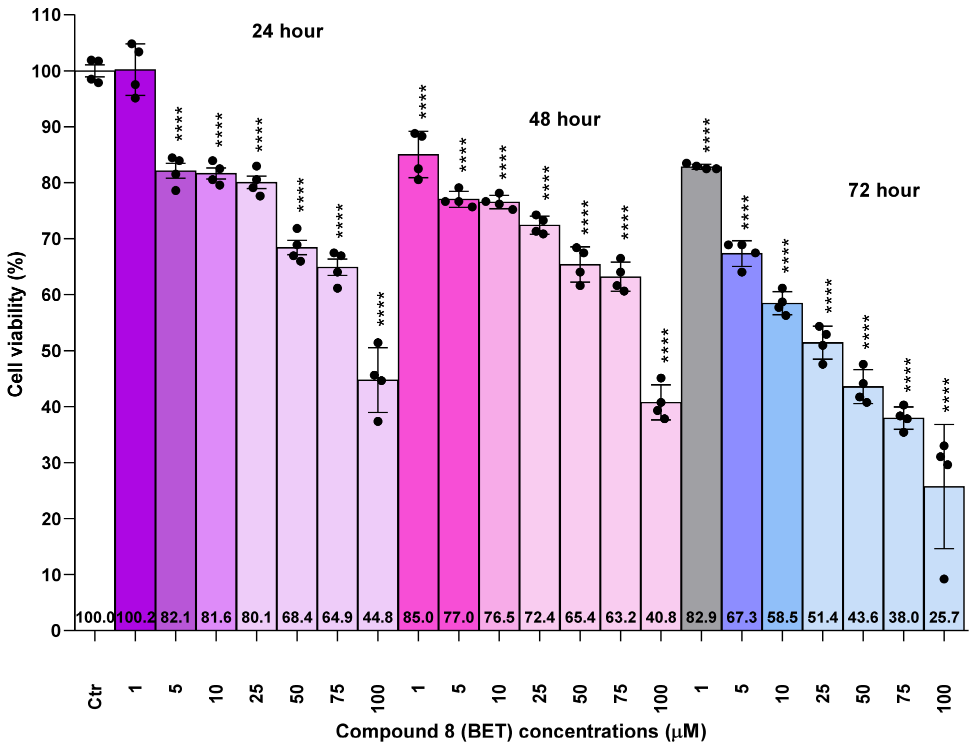
24 hour
48 hour
72 hour
Cell viability (%)
Compound 8 (BET) concentrations (μM)
Ctr 1 5 10 25 50 75 100 1 5 10 25 50 75 100 1 5 10 25 50 75 100
100.0 100.2 82.1 81.6 80.1 68.4 64.9 44.8 85.0 77.0 76.5 72.4 65.4 63.2 40.8 82.9 67.3 58.5 51.4 43.6 38.0 25.7

(O)

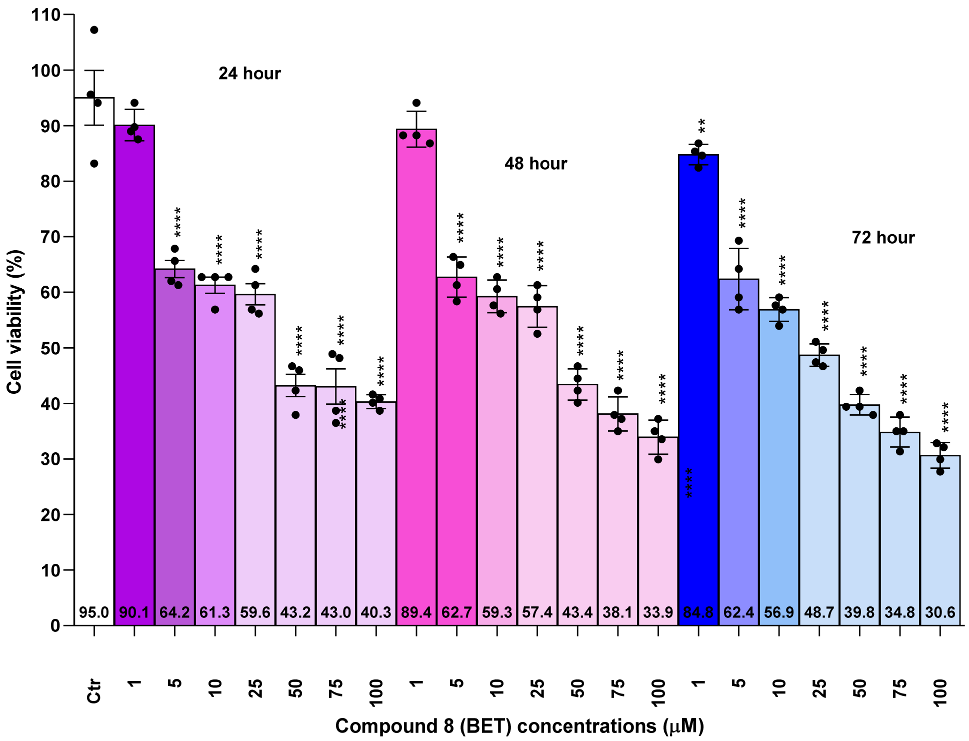
24 hour
48 hour
72 hour
Cell viability (%)
Compound 8 (BET) concentrations (μM)
Ctr 1 5 10 25 50 75 100 1 5 10 25 50 75 100 1 5 10 25 50 75 100
95.0 90.1 64.2 61.3 59.6 43.2 43.0 40.3 89.4 62.7 59.3 57.4 43.4 38.1 33.9 84.8 62.4 56.9 48.7 39.8 34.8 30.6

(P)

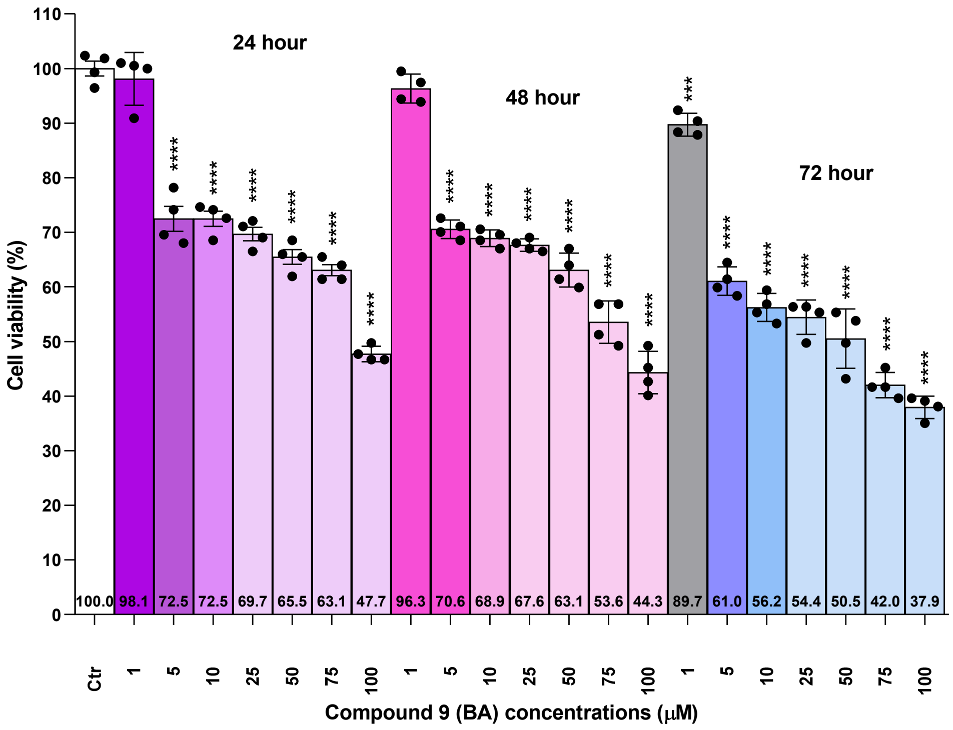
24 hour
48 hour
72 hour
Cell viability (%)
Compound 9 (BA) concentrations (μM)
100.0
98.1
72.5
72.5
69.7
65.5
63.1
47.7
96.3
70.6
68.9
67.6
63.1
53.6
44.3
89.7
61.0
56.2
54.4
50.5
42.0
37.9

(Q)

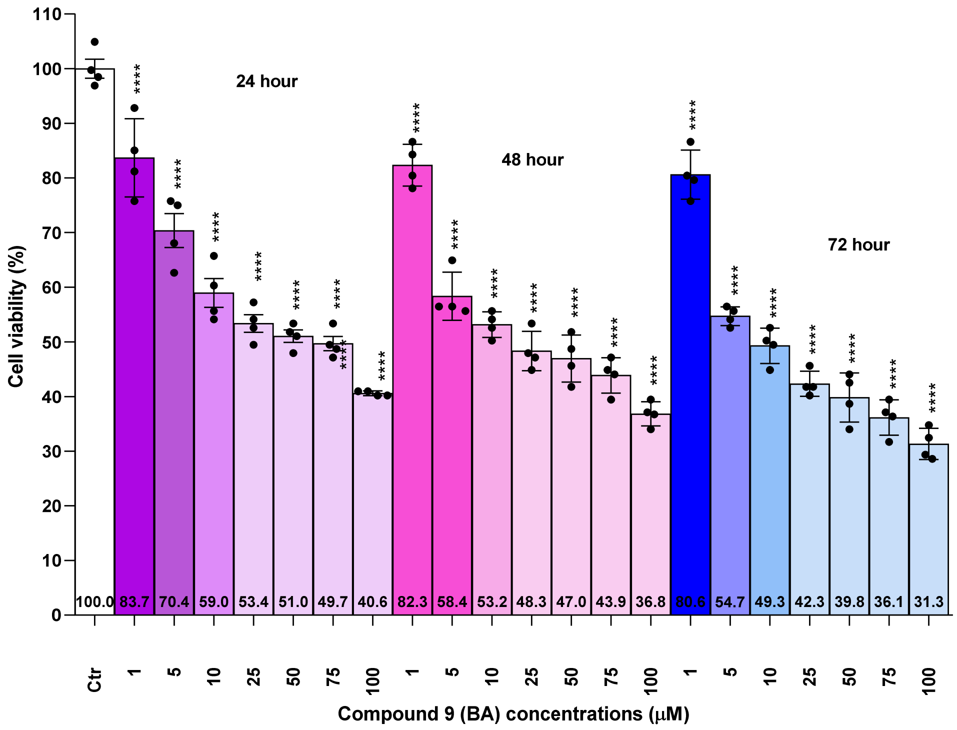
24 hour
48 hour
72 hour
Cell viability (%)
Compound 9 (BA) concentrations (μM)
100.0
83.7
70.4
59.0
53.4
51.0
49.7
40.6
82.3
58.4
53.2
48.3
47.0
43.9
36.8
80.6
54.7
49.3
42.3
39.8
36.1
31.3

(R)

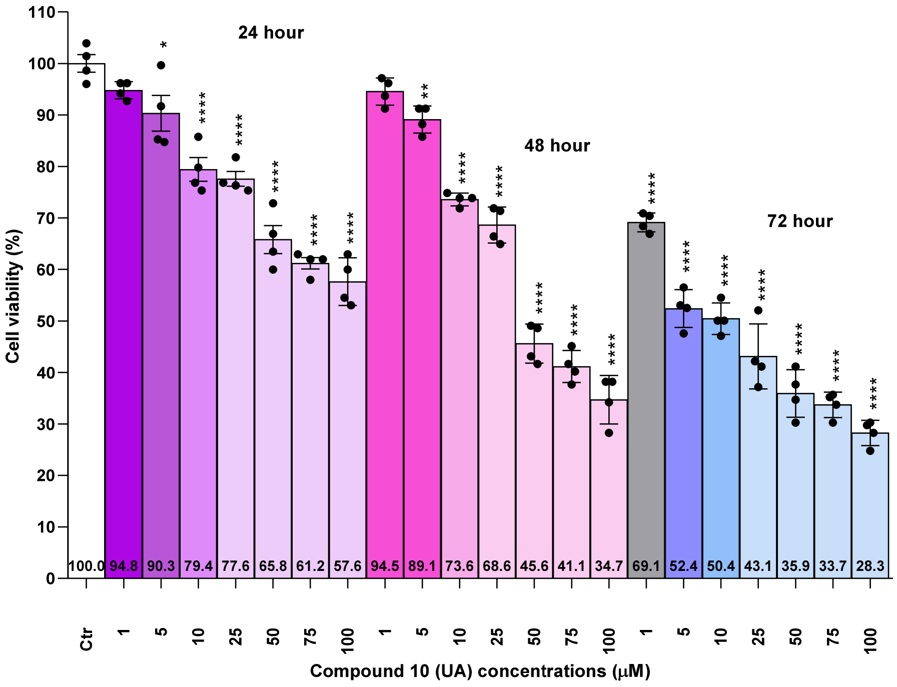

(S)

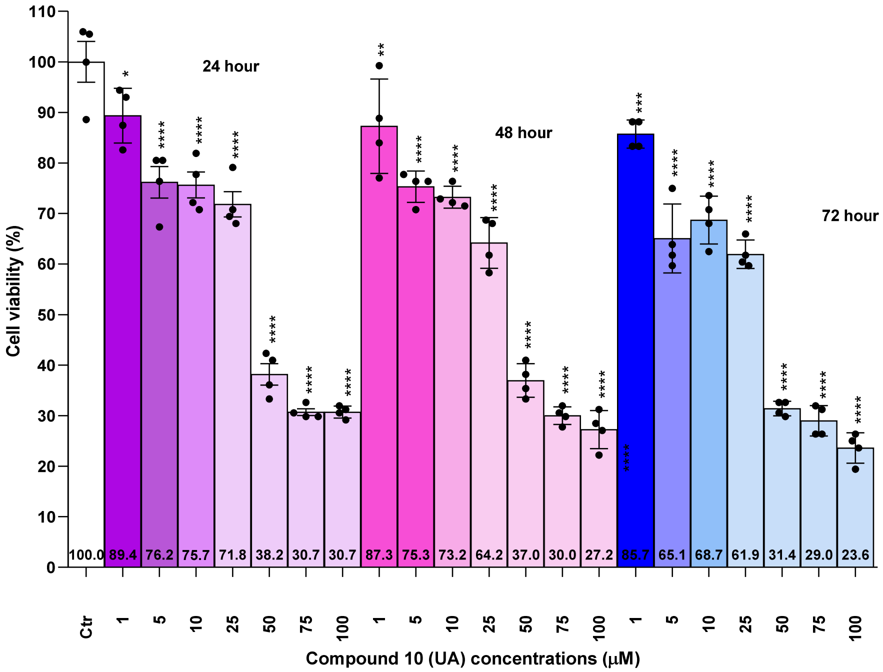

(T)

Figure S7. Cell viability was evaluated in HTLA 230 (A, C, E, G, I, K, M, O, Q, S) and HTLA ER (B, D, F, H, J, L, N, P, R, T) HR-NB cells exposed to increasing concentrations of **1** (A, B), **2** (C, D), **3** (E, F), **4** (G, H), **5** (I, J), **6** (K, L), **7** (M, N), **8** (O, P), **9** (Q, R), **10** (S, T) (1–100 μM) for 24, 48 and 72 h. Bar graphs summarize quantitative data of the means ± S.D. of four independent experiments (black spheres) run in triplicate. Significance refers exclusively to control (Ctr) using * symbol. Specifically, $p > 0.05$ no symbols; **** $p < 0.0001$; *** $p < 0.001$; $p < 0.01$ **; $p < 0.05$ * (one-way ANOVA followed by Dunnet's multi-comparisons test). Numbers within bars at bottom indicate cell viability (%).

By observing the cytotoxic profiles of compounds **1**-**3** in Figure S8A-F, differently of other cases, and especially against HTLA 230, already in 24 hours experiments, a rapid decrease in cell viability (up to < 50%, compounds **1**, **2**) at minimal concentrations (1-5 μM) was followed by unexpected peaks of increased viability up to 66.7 (**1**), 86.4 (**2**) and even 90.1% (**3**) at 25 μM, in turn followed by a new decrease (≈ 40, 50 and > 60%), to reach stability at the max concentration of 100 μM. Phenomena of increased viability (%) at the lowest concentration of 1 μM were also observed, mainly for compound **3**, between increasing time treatments. Particularly, progressive increasing in cell viability was detected at 1 μM concentrations, passing from 24- (82.9%), to 48/72-hours (101.6/95.8%). Moreover, the unexpected profiles in cells viability (%) observed in 24-hour treatments, were even more evident in 48- and especially 72-hours treatments, were fluctuating data led to values of cell viability up to even > 100% also at 75 μM, after observed cell viability of 81 (**1**), 75 (**2**) and 69 (**3**) %, associated with those already observed at 25 μM (Figure S8A, C, E). An irregular trend, sometimes at major extent and sometime softer was also observed on HTLA ER (Figure S8B, D, F).

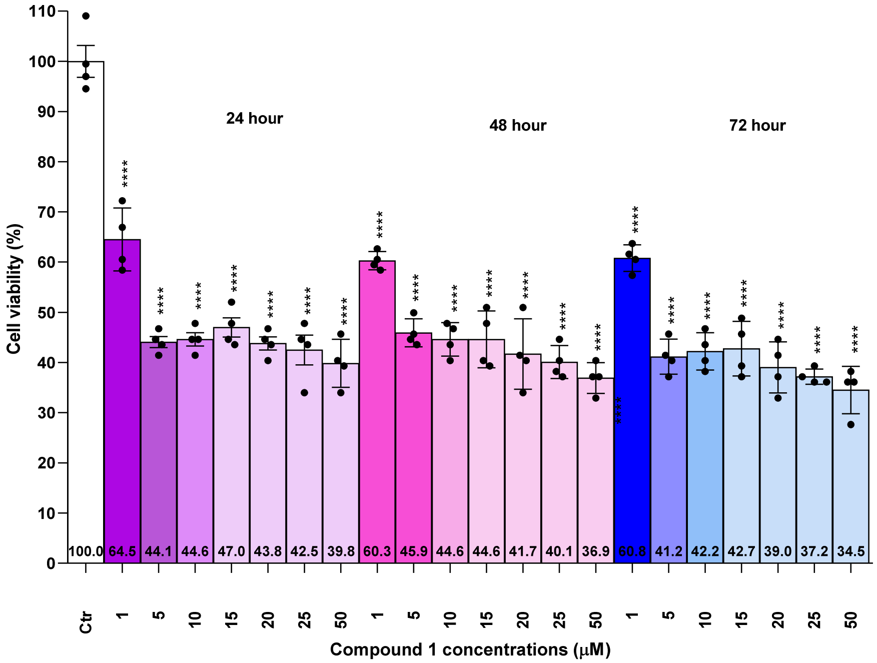
Cell viability (%)
24 hour
48 hour
72 hour
100.0 64.5 44.1 44.6 47.0 43.8 42.5 39.8 60.3 45.9 44.6 44.6 41.7 40.1 36.9 60.8 41.2 42.2 42.7 39.0 37.2 34.5
Ctr 1 5 10 15 20 25 50 1 5 10 15 20 25 50 1 5 10 15 20 25 50
Compound 1 concentrations (μM)

(A)

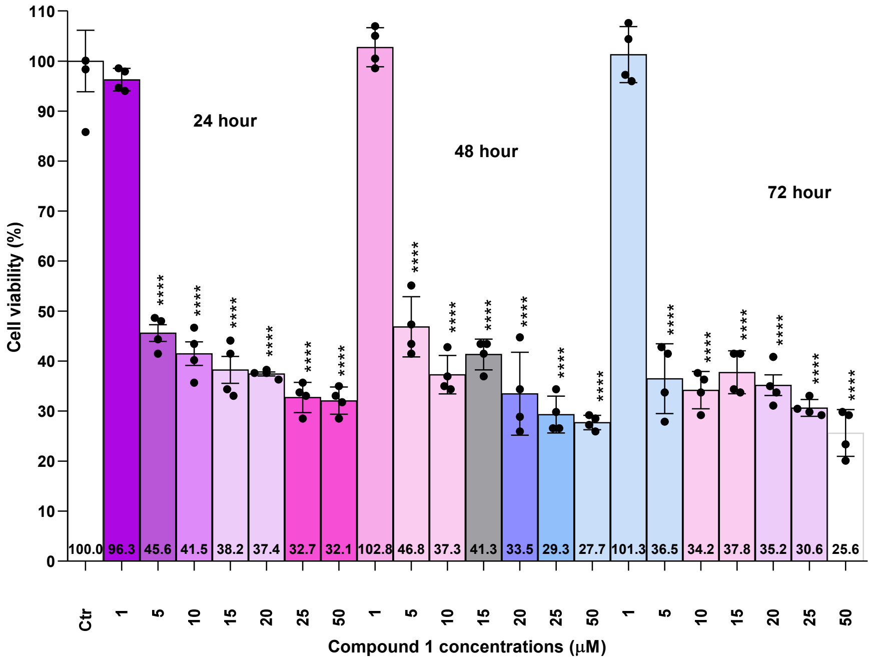
Cell viability (%)
24 hour
48 hour
72 hour
100.0 96.3 45.6 41.5 38.2 37.4 32.7 32.1 102.8 46.8 37.3 41.3 33.5 29.3 27.7 101.3 36.5 34.2 37.8 35.2 30.6 25.6
Ctr 1 5 10 15 20 25 50 1 5 10 15 20 25 50 1 5 10 15 20 25 50
Compound 1 concentrations (μM)

(B)

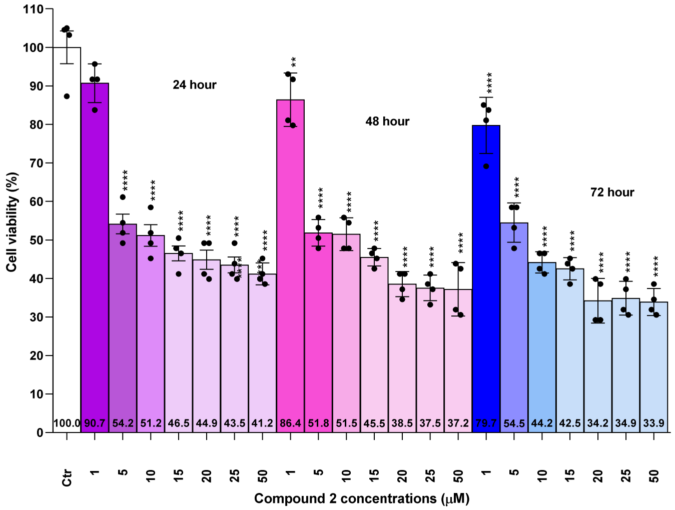
Cell viability (%)
24 hour
48 hour
72 hour
100.0 90.7 54.2 51.2 46.5 44.9 43.5 41.2 86.4 51.8 51.5 45.5 38.5 37.5 37.2 79.7 54.5 44.2 42.5 34.2 34.9 33.9
Ctr 1 5 10 15 20 25 50 1 5 10 15 20 25 50 1 5 10 15 20 25 50
Compound 2 concentrations (μM)

(C)

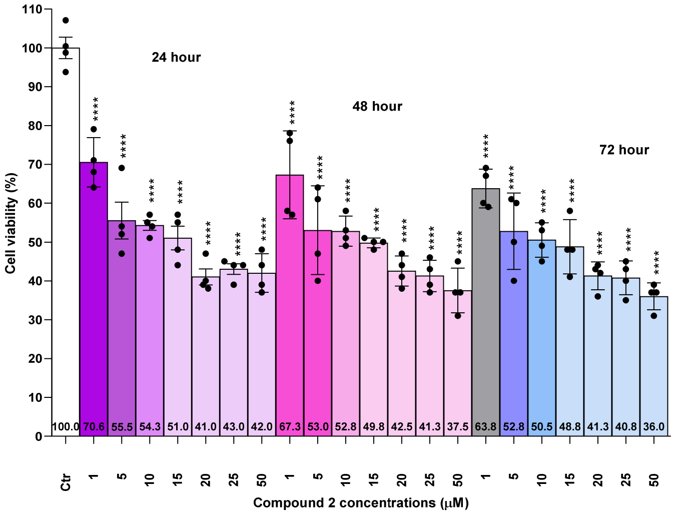
Cell viability (%)
24 hour
48 hour
72 hour
100.0 70.6 55.5 54.3 51.0 41.0 43.0 42.0 67.3 53.0 52.8 49.8 42.5 41.3 37.5 63.8 52.8 50.5 48.8 41.3 40.8 36.0
Ctr 1 5 10 15 20 25 50 1 5 10 15 20 25 50 1 5 10 15 20 25 50
Compound 2 concentrations (μM)

(D)

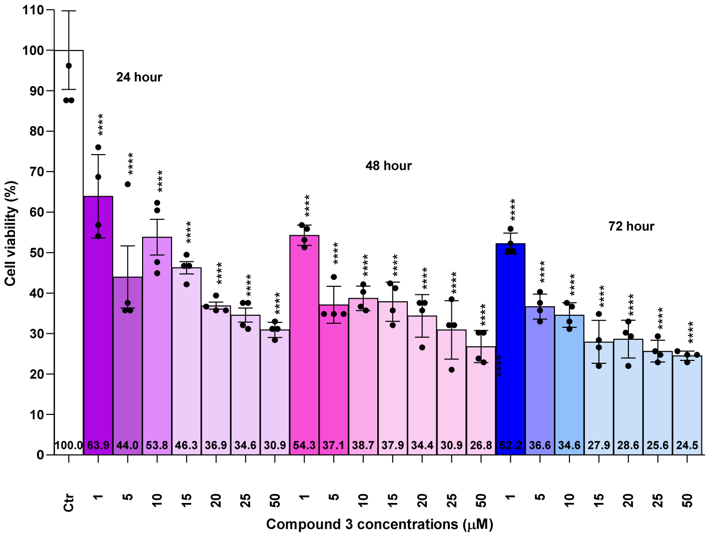
Cell viability (%)
24 hour
48 hour
72 hour
100.0 63.9 44.0 53.8 46.3 36.9 34.6 30.9 54.3 37.1 38.7 37.9 34.4 30.9 26.8 52.2 36.6 34.6 27.9 28.6 25.6 24.5
Ctr 1 5 10 15 20 25 50 1 5 10 15 20 25 50 1 5 10 15 20 25 50
Compound 3 concentrations (μM)

(E)

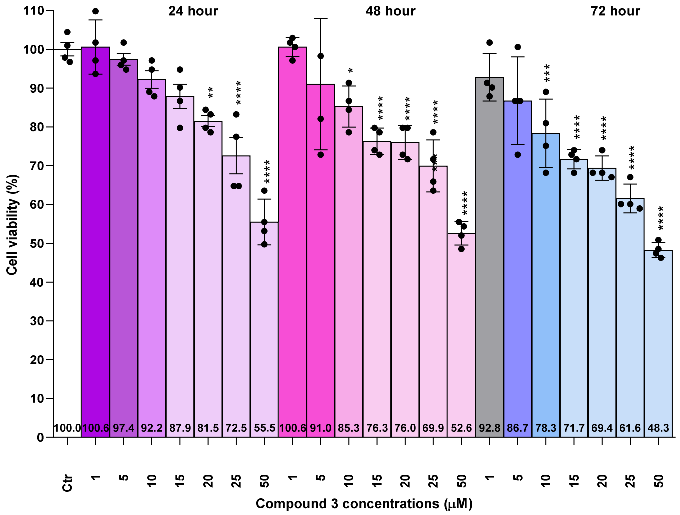
Cell viability (%)
24 hour
48 hour
72 hour
100.0 100.6 97.4 92.2 87.9 81.5 72.5 55.5 100.6 91.0 85.3 76.3 76.0 69.9 52.6 92.8 86.7 78.3 71.7 69.4 61.6 48.3
Ctr 1 5 10 15 20 25 50 1 5 10 15 20 25 50 1 5 10 15 20 25 50
Compound 3 concentrations (μM)

(F)

Figure S8. Cell viability was evaluated in HTLA 230 (A, C, E) and HTLA ER (B, D, F) HR-NB cells exposed to increasing concentrations of **1** (A, B), **2** (C, D) and **3** (E, F) (1–50 μM) for 24, 48 and 72 h. Bar graphs summarize quantitative data of the means ± S.D. of four independent experiments (black spheres) run in triplicate. Significance refers exclusively to control (Ctr) using * symbol. Specifically, $p > 0.05$ no symbols; **** $p < 0.0001$; *** $p < 0.001$; $p < 0.01$ **; $p < 0.05$ * (one-way ANOVA followed by Dunnet's multi-comparisons test). Numbers within bars at bottom indicate cell viability (%).

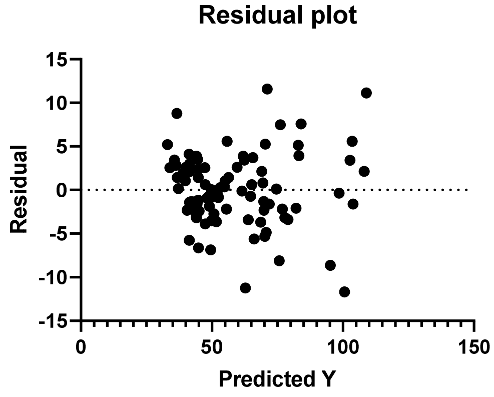

(A)

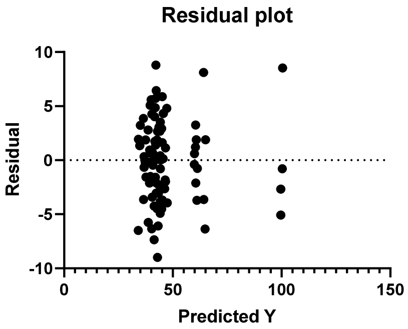

(B)

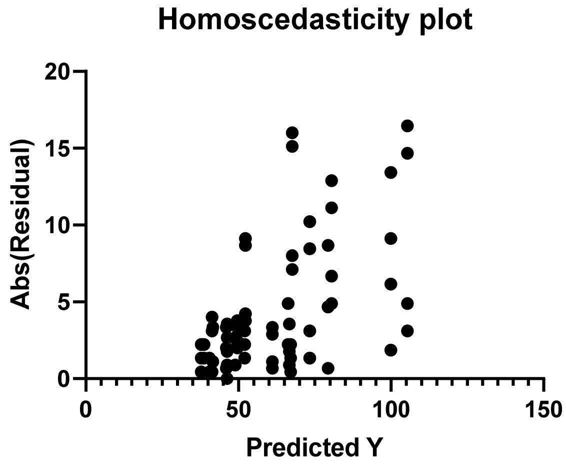

(C)

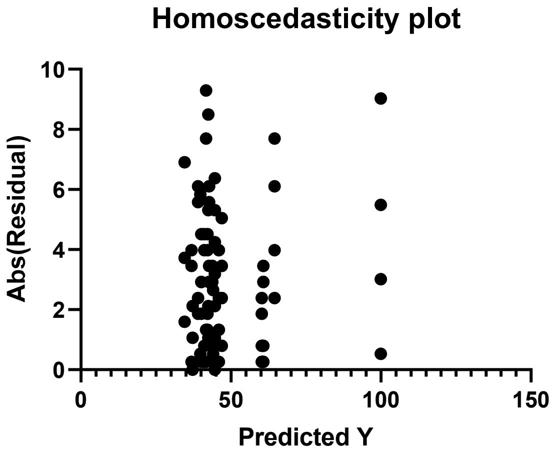

(D)

Figure S9. (A, B). Common residual plots for early (A) and second (B) MTT essays for compound **1**; (C, D) Homoscedasticity plots for early (C) and second (D) MTT essays for compound **1**.

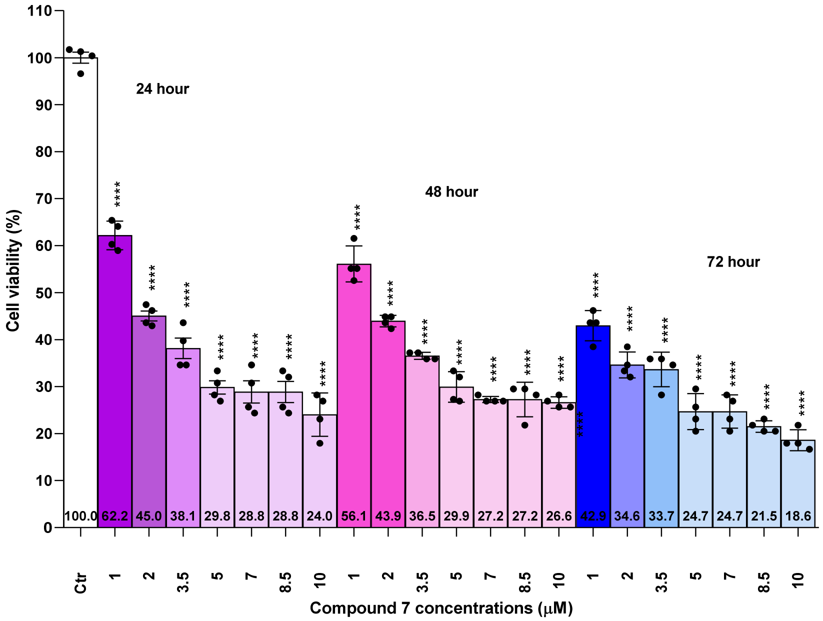

(A)

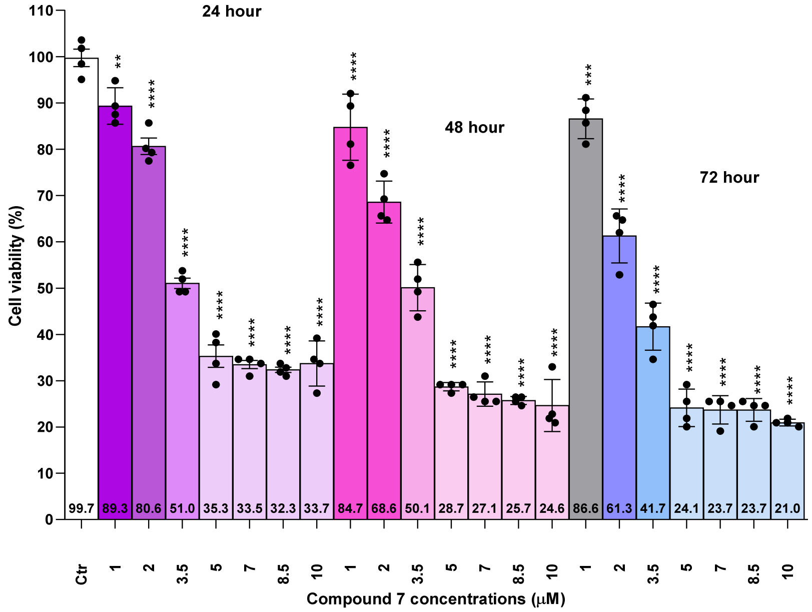

(B)

Figure S10. Cell viability was evaluated in HTLA 230 (A) and HTLA ER (B) HR-NB cells exposed to increasing concentrations of **7** (1–10 μM), for 24, 48 and 72 h. Bar graphs summarize quantitative data of the means ± S.D. of four independent experiments (black spheres) run in triplicate. Significance refers exclusively to control (Ctr) using * symbol. Specifically, $p > 0.05$ no symbols; **** $p < 0.0001$; *** $p < 0.001$; $p < 0.01$ **; $p < 0.05$ * (one-way ANOVA followed by Dunnet's multi-comparisons test). Numbers within bars at bottom indicate cell viability (%).

S2.1.1. Detailed Discussion on Observed Cell Viability Percentages as Functions of Compounds Concentrations.

In Figure S8, panels C (HTLA 230), showing behaviour of compound **2** against HTLA 230, a standout detail, sheared also by compounds **2**, **3**, despite less evident, consisted in a rapid decrease in cell viability up to < 50% at 5 μM concentration after very poor (compound **2**, viable cells 90.7, 86.4, 79.7%, depending on time treatment), or mediocre (compounds **1** and **2**, viable cells 60.8-52.2%) cytotoxic effects at the lowest concentration of 1 μM. No substantial changes occurred for increasing concentrations up to 50 μM, where viable cells were in the ranges 39.8-34.5, 41.2-33.9 and 30.9-24.5% (compounds **1**, **2** and **3**, respectively), depending on time exposure. HTLA ER cells behaved in similar way and were significantly more tolerant only to compound **3**, which displayed a weak concentration dependent cytotoxicity, slightly dependent on time treatments. Concerning compounds **1** and **2**, a behaviour like that observed against HTLA 230 for compound **2** was observed for compound **1** against ER, while for compound **2** was observed a viability profile like that observed for compound **1** against HTLA 230. Experiments of cell viability on compound **4** (Figure S7G, H) demonstrated that it was the worse performant compound against HTLA 230 cells (Figure S7G), with very poor cytotoxicity slightly dependent on concentration and time of treatments. Cell viability did not decrease under 50%, and at the highest concentration of 100 μM cell viability was 79.8, 83.1 and 71.0%, depending on time of exposure, thus establishing the inactivity of **4** on HTLA 230 phenotype. HTLA ER cells were significantly more sensitive to **4**, despite their MDR profile, showing a behaviour like that of **1** (Figure S8B), with no activity at the minimum concentration of 1 μM (99.6, 102.4, 98.2% cell viability), which rapidly converted to strong activity at the subsequent concentration of 5 μM, when cell viability was halved (56.0, 53.9, 47.8%). No further remarkable changements occurred for increasing concentrations, and a slight decreasing was observed, reaching cell viability values of 42.3, 39.0 and 36.7% at 100 μM concentration. Compounds **5** and **6** appeared significantly less potent than **1**, **2** and **3** against HTLA 230, with a profile more like that of **4**, despite a slightly higher cytotoxic activity (Figure S7I-K). Both exhibited weak concentration-dependent cytotoxic activity, capable to decrease cell viability under 50% only at 100 μM concentrations and lone in treatments of 72 hours (**6**) or 48 and 72 hours (**5**). Conversely, like compound **4**, both compounds demonstrated major cytotoxic effects against ER cells, displaying up to potent anticancer activity, thus meeting the main scope of this study. Displaying profiles like those of compound **1** and **4**, after encountering a refractory response at 1 μM concentrations (viable cells 96.7, 89.2, 75.6 (**5**) and 94.7, 91.3, 90.4 % (**6**), depending on time exposure)), both were capable to cause a cell viability < 40% immediately at 5 μM concentration. Trivial changes were then observed for higher concentrations, up to cell viability in the range 23.2-34.1%, depending on time of treatments, being compound **5** the less tolerated (Figure S7J). Unequivocally, the best anticancer compound of the mini library considered here, was the UA derivative **7**, strongly active against HTLA 230 and ER phenotypes. Specifically, compound **7** was capable to reduce cell viability of HTLA 230 under 30%, already at 5 μM administration, with trivial changes for higher concentrations, up to cell viability of 24.0, 26.6 and 18.6 already at 10 μM concentrations (Figure S10A), as well as of 23.0, 19.2 and 15.3% at 100 μM (Figure S7M, N), depending on time of treatments. The same trend was observed against HTLA ER with cell viability around 30% and < 30% at 5μM concentrations in 24 and 48/72h treatments (Figure S10B), without substantial changes for increasing values. Particularly, **7** encountered substantial recalcitrance in ER phenotype, only at the lowest concentrations of 1 μM (Figure S7N) or 1, 2 μM (Figure S10B), thus making its cytotoxic profile resembling those of compound **1**, **4**, **5** and **6** against ER and **2**, against HTLA 230. In this regard, compound **7** could represent a very promising molecule to develop a multitarget therapeutic capable to exterminate in comparable way both phenotypes of very aggressive metastatic HR-NB. Pristine BET, BA and UA were generally less performant of derivatives, as widely reported 54], being BET the better tolerated compound by both cell phenotypes, despite HTLA ER phenotype appeared more sensitive than HTLA 230 one to all natural triterpenoids.

Table S1. *In vitro* IC_{50} * anticancer activity thresholds with associated anticancer potency.

Activity Category	Typical IC_{50} Range	Interpretation	Refs
Strongly active	< 1 μM	High potency, typical of the most promising lead compounds	[54,55]
Potent/Good activity	1–10 μM	Significant activity, often acceptable for hit/lead compounds	[55]
Moderately active	>10 to 50 μM	Limited activity; interesting only if highly selective	[56,57]
Generally inactive	> 50 μM	Usually not considered a viable anticancer candidate	[58,59]

* IC_{50} is a standard measure of potency in inhibition assays.

Table S2. IC_{50} activity thresholds for triterpene derivatives or other compounds in HTLA 230, HTLA-ER NB cells and in other cells which possess different pattern of sensitivity/resistance.

Activity category	IC_{50} range (μM)	Interpretation	Refs
Strongly active	< 1	↑ Potent; rare for TTPs unless optimized derivatives; NB cells can show IC_{50} < 1 μM to ↑↑ cytotoxic agents	[61]
Potent/Good activity	1–10	Typical IC_{50} range for TTPs in low aggressive NB cells [65]; Biologically meaningful compounds for hit/lead progression	[11,63]
Moderately active	> 10 to 50	Compounds with moderate relevance ** Typical for resistant NB cells, being derived from metastatic bone marrow	[63]
Generally inactive	> 20–50	Not considered active for anticancer development, especially in NB studies unless targeting non-cytotoxic pathways	[64]

* Including MDR variants, HTLA 230 and ER; **unless the compounds show exceptional selectivity or mechanistic novelty; ↑ = highly, potent; TTPs = triterpenes.

S2.1.2. Ranked List of Different Neuroblastoma Cell Phenotypes

In this Section, using literature data for different NB cell lines, a ranked list of wile-type NB cell lines (from easiest to hardest to counteract), has been provided, based on their intrinsic biological aggressiveness, heterogeneity, cellular adaptability, tumorigenic capacity, natural resistance and tendency to develop resistance under chronic treatments. Resistant or even MDR counterparts of these wild phenotypes represent NB forms, even more difficult to defeat, following the same hierarchy. Specifically, the differentiable cell line SH-SY-5Y, deriving from SK-N-SH one, is among the cell lines easier to inhibit. It is not particularly associated with aggressive phenotypes, having low intrinsic aggressivity [67,68]. ATCC CRL-2266 SH-SY-5Y NB cells are not considered 'aggressive' or 'resistant' in the classical oncological sense, and possess specific biological features, that make them more sensitive to drugs stress, depending on the treatment [69-71]. Moreover, direct comparison with other cell lines demonstrated that IMR-32, SK-N-BE and generically MYCN amplified lines such HTLA 230 cells, used by us, are more aggressive than SH-SY-5Y cells, which are frequently used by authors. The heterogeneous parental line SK-N-SH deriving by medullar metastasis, containing mixed neuroblastic (N-type) and substrate-adherent (S-type) cells, can be retained slightly more aggressive than SH-SY-5Y. Anyway, lacking MYCN amplification, this line is historically considered (*in vitro*) less aggressive than MYCN-amplified lines, such as HTLA 230 cells and endowed with moderate aggressivity [72,73]. SK-N-AS is a subclone of SK-N-SH, deriving by bone metastasis, presenting S-type (substrate-adherent) phenotype, muted p53 gene and resistant to 6-thioguanine. It is known to be a more proliferative, less differentiated, more tumorigenic, and aggressive phenotype, respect to SK-N-SH one, but not MYCN-amplified. While SK-N-SH cells form moderately aggressive tumours in xenograft models, SK-N-AS generally displays greater growth and invasiveness. SK-N-SA are less responsive to differentiation and maintains a more "stem-like" profile, associated with greater aggressiveness [https://www.cytion.com/About-Cytion/Knowledge-Hub/Blog/Proteome-Profiling-of-SK-N-AS-Neuroblastoma-Cells, accessed on 06 May 2026] [74]. However, between SK-N-AS and HTLA 230, HTLA 230 remains the most difficult lineage to defeat in terms of biological aggressiveness and tumorigenic behaviour. Not belonging to the group of MYCN-amplified NB, tumours developed by SK-N-AS cells are typically less malignant than those developed by HTLA 230. Conversely, being a MYCN-amplified lineage, HTLA 230 NB cells are strongly associated with very high-risk NB (HR-NB), characterized by rapid growth, invasiveness, and therapeutic resistance [64]. Collectively, HTLA 230 cells are frequently used as a model of metastatic, highly aggressive and refractory to treatment NB phenotype, more difficult to defeat than SK-N-AS, SK-N-SH and SH-SY-5Y ones. Since they combine MYCN amplification with intrinsic great proliferative aggressiveness, high capacity for survival, apoptosis evasion, and plasticity, as well as high genetic aggressiveness [73,75] and refractory behaviour to several drugs, HTLA 230, even before developing MDR, possess the typical phenotype of high tolerant HR-NB, with very high level of malignancy [64]. The MDR HTLA ER selected by HTLA 230 wild phenotype with their chronic/high concentration treatment using ETO over six months, are extensively more difficult to inhibit [75]. IMR-32 cells generally display malignancy higher than that of HTLA 230 phenotype but being a MYCN-amplified very intrinsically aggressive and highly tumorigenic line *in vivo*, IMR-32 phenotype tends to form tumours rapidly and is particularly suitable as NB model for *in vivo* experiments [73,76]. However, HTLA ER phenotype, become resistant to more than one existing clinically used chemotherapeutics, is even more difficult to be treated *in vitro* than IMR-32 [6]. Their MDR forms (HTLA-ER cells) represent the most difficult to be biologically controlled and therapeutically combated NB cell lines [66]. Genetic alterations and metabolic adaptations such as 13q14.3 deletion, miR-15a/16-1 downregulation and increased BMI-1, make HTLA ER cells very robust to chemotherapeutic stress [77].

Table S3. Classification of cytotoxic potency of compounds **1-10** against sensitive HTLA-230 and MDR HTLA ER NB cells at 24, 48 and 72-hours exposure according to data in Table S1 and S2.

TPs	Time (h)	HTLA 230		HTLA ER	
		IC_{50} (μM)	Potency	IC_{50} (μM)	Potency
	24 h	5.99	Potent	8.23	Potent
**1**	48 h	4.42	Potent	8.07	Potent
	72 h	3.29	Potent	6.71	Potent
	24 h	13.96	Medium	12.65	Medium
**2**	48 h	10.70	Medium	9.95	Good
	72 h	8.11	Potent	8.02	Potent
	24 h	5.47	Potent	56.79	Low/inactive
**3**	48 h	1.53	High	56.48	Low/inactive
	72 h	1.17	Very high	47.76	Medium/Low
	24 h	328.5	Inactive	31.13	Medium/Low
**4**	48 h	394.3	Inactive	23.49	Medium/Low
	72 h	202.9	Inactive	16.5	Medium
	24 h	105.50	Inactive	12.89	Medium
**5**	48 h	68.87	Inactive	9.51	Good
	72 h	35.12	Medium/Low	6.38	Potent
	24 h	84.67	Inactive	11.60	Medium
**6**	48 h	75.80	Inactive	9.55	Good
	72 h	65.25	Low/inactive	7.48	Potent
	24 h	1.75	Very high	4.30	High/Very high
**7**	48 h	1.38	Very high	3.39	High/Very high
	72 h	0.58	Exceptional	2.89	High/Very high
	24 h	98.28	Inactive	35.79	Medium/Low
**8**	48 h	79.54	Inactive	29.97	Medium/Low
	72 h	25.87	Medium/Low	22.71	Medium/Low
	24 h	82.44	Inactive	39.99	Medium/Low
**9**	48 h	65.22	Inactive	26.98	Medium/Low
	72 h	33.06	Medium/Low	17.06	Medium
	24 h	106.00	Inactive	37.17	Medium/Low
**10**	48 h	46.61	Low	31.91	Medium/Low
	72 h	14.68	Medium	25.49	Medium/Low

Table S4. Comparison of cytotoxicity, in terms of IC_{50} µM concentrations, of compounds **1**-**7** against both HTLA 230 and ER NB cells at 24, 48 and 72 hours treatments, respect to that of their natural sources (compounds **8**, **9** and **10**).

Compound	HTLA 230			HTLA ER		
	24 hours	48 hours	72 hours	24 hours	48 hours	72 hours
**1 (8)**	↑	↑	↑	↑	↑	↑
**2 (8)**	↑	↑	↑	↑	↑	↑
**3 (9)**	↑	↑	↑	↓	↓	↓
**4 (9)**	↓	↓	↓	↑	↑	↑
**5 (8)**	↓	↑	↓	↑	↑	↑
**6 (8)**	↑	↑	↓	↑	↑	↑
**7 (10)**	↑	↑	↑	↑	↑	↑

In bold green BET (**8**) triterpenoids derivatives: **1**, **2**, **5** and **6**; in bold red BA (**9**) triterpenoids derivatives: **3** and **4**. Compound **7** (bold light blue) is an UA (**10**) derivative. ↓ means a decreased activity respect pristine compounds regardless the overall intrinsic potency; ↑ means an increased activity respect pristine compounds, regardless the overall intrinsic potency.

In Table S4., arrow icons in purple indicate cases in which the anticancer activity of natural triterpenes was ameliorated (33 out of 42), i.e. 79%. Respect to their source BET, compounds **1** and **2** demonstrated an ameliorated activity in all-time treatments against both cell lines. The reproduction of their synthesis, to carry out other investigations, would be rationally convenient for their potential development and clinical translation as new anticancer devices against HR-NB cells, including the challenging ER cell line, no longer sensitive to all available antitumour drugs. Compound **3** overperformed BA at all time exposure against HTLA 230, while underperformed BA against HTLA ER cells, demonstrating very poor activity. Compound **4**, different from **3** only for the presence of 6-TPP-hexanoic acid cationic residue in C-3, demonstrated a cytotoxic behaviour opposite to that of **3**, outperforming BA against HTLA ER, and being completely inactive against HTLA 230. Compounds **5** and **6** displayed some improvements to the activity of pristine BET against HTLA 230 cells, depending on time of exposure, but displaying collectively anticancer inactivity. Anyhow, both compounds strongly improved BET activity at all time exposure against HTLA ER NB cells, reaching time dependant IC_{50} values in low micromolar ranges, thus establishing for a very good cytotoxic activity. On this scenario, their synthesis and further development could be rationally convenient to exterminate critical HR-NB ER cells, no longer treatable differently. Compound **7** always overperformed UA activity against both cell lines and at all times-exposure tested, displaying IC_{50} considered exceptional for triterpenes, in 72-hs treatments. Therefore, the reproduction of its synthesis for further investigations and future clinical translation is advisable, convenient and welcome.

S2.1.3. Further Comparisons Between Anticancer Potency of Our Best **1**, **2**, **3** and **7** Against HTLA and that Already Reported for Analogous Compounds

Despite not triterpenoids-based compounds, Nakamura et al identified seven small-molecules (A–G) capable of targeting the BDNF-binding domain of TrkB and inducing apoptosis in NB cells. Specifically, A-G showed an in vitro potent cytotoxicity in TrkB-expressing NB cell lines [86], which are a MYCN-amplified NB cell line derived from a tumour of the left adrenal area of an 18-month-old male patient, consisting of adherent colonies comprising neuroblast-like cells forming dense mounds. This cell line has been reported to have tyrosine hydroxylase activity [91]. IC_{50} values ranged from 0.07 to 4.6 µM against CHP134 cells were observed [86]. Similar IC_{50} values and inhibition processes were observed in not MYCN-amplified SH-SY-5Y/TrkB cells and in xenograft models without detectable toxicity [86]. Before comparing results obtained for triterpenes derivatives **1**-**7**, with those reported by Nakamura et al., it must be considered that authors used different proliferation essays of days respect to our metabolic tests (MTT) of max 72 hours to assess the cytotoxic effects

of A-G. Additionally, despite CHP-134 is also a highly malignant, MYCN-amplified NB cell line, with an aggressive metastatic HR-NB-like profile of HTLA 230 cells, HTLA-ER ones are more aggressive and therapy-resistant, since they add acquired MDR and metabolic adaptations, encompassing 13q14.3 monoallelic deletion with miR-15a/16-1 downregulation, leading to apoptosis evasion and BMI-1 upregulation, reducing p16 and promoting survival and metabolic adaptation, making it biologically harder to contro l [3]. On these considerations, compounds **3** and **7** showing 1.17and 1.10 μM IC_{50} respectively (72h) on HTLA-230, demonstrated remarkable anticancer potency, higher than that of 5 out of 7 compounds developed by Nakamura et al. by 1.2-3.9- and 1.3-4.2-fold. Moreover, compound **1** displaying 3.29μM IC_{50} (72h), demonstrated higher anticancer potency than compound D, developed by Nakamura et al., by 1.4-fold. Additionally, despite against MDR HTLA ER phenotype unequivocally more difficult to defeat than not resistant CHP134 NB cells, compound 7 (IC_{50} = 4.16 μM) was more potent of compound D, while compounds **1**, **5** and **6** showed an anticancer potency equivalent. In the field of triterpene derivatives, but assayed against cancer cells different from NB ones, Spivak et al reported the design, synthesis, and biological evaluation of a series of mitochondria-targeting pentacyclic triterpenoids, obtained by conjugating BET, BA, ursolic, oleanolic, and glycyrrhetinic acids with the delocalized lipophilic cation F16 [20]. As observed in the present study, for compounds **1**, **4**, **5**, **6** and **7**, possessing the mitochondria-targeting TPP lipophilic cation, F16–triterpenoid lipophilic cationic conjugates developed by Spivak, showed remarkable increases in cytotoxic potency of related parent triterpenoids [20], thus confirming our findings. In the same year, the same authors published a collection of articles concerning the anticancer effects of cationic triterpenoids derivatives, prepared by their chemical modification with F16, TPP^+, rhodamine$^+$, against various types of tumour cells including melanoma, adenocarcinoma, neuroblastoma, medulloblastoma, and glioblastoma, all evidencing strong improvements of the anticancer activity of pristine natural compounds [88]. Yalçınkaya et al demonstrated that BA can induce mitochondrial apoptosis and reduced cell viability up to 62.0% (24 hours) and 42.0% (48 hours) at the maximum concentration tested of 80 μM in SH-SY-5Y, by upregulating pro-apoptotic genes (Bax, Caspase-3, p53, p21) and downregulating anti-apoptotic and proliferation-related genes (Bcl-2, CDK1, Cyclin B1, Ki-67, EAG1) [82]. Specifically, IC_{50} values in 48 hours experiments were 75 μM confirming our findings on HTLA 230 (IC50 = 65.22 μM). Concerning semisynthetic derivatives of BA by Suresh et al, the developed new ionic derivatives of BA showed much higher inhibitory effects than BA against different cancer cell lines, including SH-SY-5Y NB cells [80]. The best performant compound developed by Suresh et al displayed IC_{50} values = 67.5 μM (48-hours experiments). In this regard, BA-derivative **3** reported here, despite poorly active against MDR HTLA ER NB cells no longer treatable with available drugs, demonstrated IC_{50} values lower by 1.2, in 48-hours experiments [80]. Conversely, when tested against HTLA 230 NB cells, strongly more aggressive and recalcitrant to treatments than SH-SY-5Y cells, demonstrated an anticancer potency 44.1-times higher [80]. Finally, glycyrrhetinic acid, which is an oleananic triterpene different from *cores* here reported and used by us, was strongly chemically modified by Alho et al, to achieve C-2 heterocyclic triterpene derivatives (compounds **9**-**14**, **17**, **18**, **21**, **22**) [81]. In 72-hours experiments against SH-SY-5Y NB cells compounds **9**-**12**, **21** and **22** displayed IC_{50} values of 1.7, 2.2, 3.2, 3.7, 5.6 and 6.0 μM. In this regard, compounds **3** and **7** developed here demonstrated an anticancer potency against MYCN-amplified HTLA 230 cells higher by 1.5-5.1 and 1.5-5.5-times, respectively. Moreover, against the same cells, our compound **1** was more potent than compounds **9**, **11** and **21**, synthesized by Alho et al, by 1.8, 1.7 and 1.1-fold, respectively. Finally, against MDR HTLA NB cells, compound **7** by us, was more potent than Alho's et al compounds **9** and **11**, by 1.3 and 1.1-times [85]. Additionally, Tabopda et al. isolated from *Elephantopus mollis* a new triterpene derivative belonging to the lupane family as BET and BA and other sesquiterpene lactones [88], which were tested against B104 NB cells, a cell line originated from the central nervous system of rats, and therefore it does not exhibit MYCN amplification, which can occur only in human NB. Despite B104 cells may be useful for neuronal studies but

is not an appropriate model for cytotoxic experiments with new compounds against NB, authors observed induction of apoptosis and from moderate to potent anticancer effects [92]. Compound **1** by Tobopda et al, which can be seen as a chemically modified derivative of BET and BA, as our compounds **1**, **2**, **5**, **6** and **3**, **4**, respectively displayed IC_{50} values = 25.2 µM. In this regard, compounds **1**, **2**, **3** developed here, when tested against HTLA 230 were more potent by 1.8-21.5-fold. Moreover, compounds **1**, **2** and **5**, **6**, when tested against HTLA ER cells, where more potent than **1** developed by Tabopda et al, by 2.0-3.7 and 2.0-3.9-fold, respectively, despite the MDR profile of ER cells [88]. Pharmacosome system with *Moringa oleifera* extract encompassing a rich blend of triterpenoids and terpenes administered to SH-SY5Y NB cells using a nanocarrier showed a ~10-fold reduction in IC_{50} respect not nano manipulated extracts, strongly enhancing anticancer activity [93]. This suggests that the already high intrinsic activity of the weak one of our compounds against HTLA 230 and ER cells, could be greatly improved by using proper delivery methods.

Table S5. Comparison of IC_{50} values of compounds **1-10**, and those of etoposide (ETO) in HTLA 230 and MDR HTLA ER neuroblastoma.

**Compound**	**IC_{50} HTLA 230 (µM)**	**Ratio IC_{50} ETO/Cs**	**IC_{50} MDR HTLA ER (µM)**	**Ratio IC_{50} ETO/Cs**
**1**	**5.99**	24.8748	**8.23**	62.0899
**2**	**13.96**	10.7163	**12.65**	40.3953
**3**	**5.47**	27.3492	56.79 *	8.9981
**4**	328.5 *	0.45540	31.13	16.4150
**5**	105.50 *	1.4180095	**12.89**	39.6431342
**6**	84.67 *	1.7668596	**11.60**	44.0517241
**7**	**1.75**	85.485714	**4.30**	118.83720
**8**	98.23 *	1.5229563	35.79 *	14.2777312
**9**	82.44 *	1.8146531	39.99 *	12.7781945
**10**	106.00 *	1.4113208	37.17 *	13.747646
**ETO**	**149.60 ***	**1.0000000**	**511.00 ***	**1.0000000**

* Extrapolation provided by the nonlinear model mentioned in the main text; more interesting results are in bold red; results from administering ETO for 24 h are in black and bold in the last row.

N.B. References in this SM are refereed to reference list after main text.
